# Inherited Follicular Epithelial-Derived Thyroid Carcinomas: From Molecular Biology to Histological Correlates

**DOI:** 10.1007/s12022-020-09661-y

**Published:** 2021-01-25

**Authors:** José Manuel Cameselle-Teijeiro, Ozgur Mete, Sylvia L. Asa, Virginia LiVolsi

**Affiliations:** 1 Department of Pathology, Galician Healthcare Service (SERGAS), Clinical University Hospital, Travesía Choupana s/n, 15706 Santiago de Compostela, Spain; 2 Health Research Institute of Santiago de Compostela (IDIS), Santiago de Compostela, Spain; 3 Medical Faculty, University of Santiago de Compostela, Santiago de Compostela, Spain; 4 Department of Pathology and Endocrine Oncology Site, University Health Network, Toronto, ON Canada; 5 Department of Laboratory Medicine and Pathobiology, Faculty of Medicine, University of Toronto, Toronto, ON Canada; 6 Department of Pathology, University Hospitals Cleveland Medical Center, Case Western Reserve University, Cleveland, OH 44106 USA; 7 Department of Pathology and Laboratory Medicine, Perelmann School of Medicine of the University of Pennsylvania, Philadelphia, PA USA

**Keywords:** Thyroid cancer, Familial non-medullary thyroid carcinoma, *APC*, *PTEN*, *SDHB*, *DICER1*, *PRKAR1A*, *WRN*, *RASAL1*, Cribriform-morular thyroid carcinoma, FAP, Cowden syndrome, PTEN-hamartoma tumor syndrome, DICER1 syndrome, Carney complex, Wermer syndrome

## Abstract

Cancer derived from thyroid follicular epithelial cells is common; it represents the most common endocrine malignancy. The molecular features of sporadic tumors have been clarified in the past decade. However the incidence of familial disease has not been emphasized and is often overlooked in routine practice. A careful clinical documentation of family history or familial syndromes that can be associated with thyroid disease can help identify germline susceptibility-driven thyroid neoplasia. In this review, we summarize a large body of information about both syndromic and non-syndromic familial thyroid carcinomas. A significant number of patients with inherited non-medullary thyroid carcinomas manifest disease that appears to be sporadic disease even in some syndromic cases. The cytomorphology of the tumor(s), molecular immunohistochemistry, the findings in the non-tumorous thyroid parenchyma and other associated lesions may provide insight into the underlying syndromic disorder. However, the increasing evidence of familial predisposition to non-syndromic thyroid cancers is raising questions about the importance of genetics and epigenetics. What appears to be “sporadic” is becoming less often truly so and more often an opportunity to identify and understand novel genetic variants that underlie tumorigenesis. Pathologists must be aware of the unusual morphologic features that should prompt germline screening. Therefore, recognition of harbingers of specific germline susceptibility syndromes can assist in providing information to facilitate early detection to prevent aggressive disease.

## Introduction

A minor proportion of thyroid tumors is caused by germline susceptibility; within this group, these tumors derive from C cells (medullary thyroid carcinoma, MTC) or from follicular cells. While about 25% of MTCs are hereditary and their genotype–phenotype relationship is well established, up to 10% of follicular epithelial-derived thyroid carcinomas are hereditary and their histological and molecular characteristics are much less well known [1–9]. Exceptionally, in some populations with many relatives living close together, a prevalence of up to 13.5% of inherited follicular cell derived carcinomas has been reported [10]. Inherited follicular epithelial derived thyroid carcinoma (TC) are usually referred to as familial non-medullary thyroid carcinomas (FNMTCs) and classified into two main subgroups depending whether the predominant cancer is the thyroid tumor (non-syndromic FNMTCs (NSFNMTCs)) or if it is a thyroid cancer that appears in a patient with a predominance of non-thyroid tumors (syndromic FNMTCs (SFNMTCs)) [9].

An important limitation in determining the true incidence of FNMTCs is related to the fact that follicular epithelial-derived thyroid carcinomas (including incidental papillary microcarcinomas) are common in the general population. A careful clinical documentation of family history or familial syndromes that can be associated with thyroid disease can help identify germline susceptibility-driven thyroid neoplasia. Thyroid neoplasms in three or more family member or the diagnosis of differentiated thyroid carcinoma with paternal inheriance in the proband may be a harbinger of inherited disease [11]. In addition, thyroid neoplasia can be the first clinically detected manifestation in some SFNMTCs as a significant number of patients with inherited FNMTCs also manifest with what appears to be sporadic disease. Therefore, detailed cytomorphologic assessment of thyroid nodules, application of molecular immunohistochemistry for relevant biomarkers, and careful assessment of the non-tumorous thyroid parenchyma can help diagnosticians in the detection of an inherited disease. From a clinicopathologic perspective, inherited follicular epithelial-derived thyroid carcinomas tend to have early onset disease with increased frequency of multifocal tumors that arise in the background of benign follicular nodular disease [11].

This article reviews the main characteristics of inherited follicular epithelial-derived thyroid carcinomas, emphasizing the genotype–phenotype correlations in a way that can be especially useful to pathologists for the recognition of the possible familial character of certain follicular cell-derived carcinomas in daily practice.

## Non-Syndromic Familial Non-Medullary Thyroid Carcinoma (NSFNMTC)

To the best of our knowledge, in 1955 David Robinson and Thomas Orr published the first description of non-syndromic familial non-medullary thyroid carcinomas (NSFNMTCs) [12]; the patients, two identical 24-year-old twin sisters, showed several foci of classic papillary thyroid carcinoma (PTC) in their thyroid lobes and lymph node metastases. As was described in this prototypical first example, NSFNMTCs are usually PTCs (> 85%) characterized by an early onset, more bilaterality and multifocality and nodal metastases [10, 13–15].

Given the lack of distinctive histological features in this group of familial thyroid tumors, it has been proposed that the clinical diagnosis of NSFNMTC should be based on the evidence of PTC in two or more first-degree relatives, or on the finding of multinodular goiter (MNG) in at least three first- or second-degree relatives of a PTC patient, of course, always in the absence of previous ionizing radiation exposure and neoplasia syndromes [16, 17]. As secondary criteria the following have been proposed: the diagnosis in a patient younger than 33 years, multifocal or bilateral PTC, organ-exceeding tumor growth, metastasis, and familial accumulation of adolescent-onset thyroid disease [16]. Due to the general predominance of PTC in women, the diagnosis of PTC in a male, particularly in a young man, is also suggestive of a familial predisposition [18, 19]. Since the probability that it is not a sporadic carcinoma rises to more than 95% when three family members are affected [2, 20], the most recent studies suggest reserving the definition of NSFNMTC for all those cases with a minimum of three first-degree relatives diagnosed with follicular cell-derived thyroid carcinomas [3, 8, 10, 20, 21].

Patients with NSFNMTC tend to be younger than those with sporadic-non-medullary thyroid carcinoma (SNMTC) [3, 5, 14, 22–25], although not all series have found significant differences [1, 4, 6, 21, 26, 27]. Clinical “anticipation” with the second generation exhibiting the disease at an earlier age and having more advanced disease at presentation has been described in these families with NSFNMTC [7, 28], although the possibility of a bias due to more frequent evaluation in the familial group than in controls has been proposed [21]. Screening of at-risk family members resulted in earlier detection of low-risk FNMTC and was associated with a less aggressive initial treatment [29]. No significant differences in gender have been found between the sporadic and familial groups with thyroid carcinoma [10, 21].

### Pathological Features

**Fig. 1:**
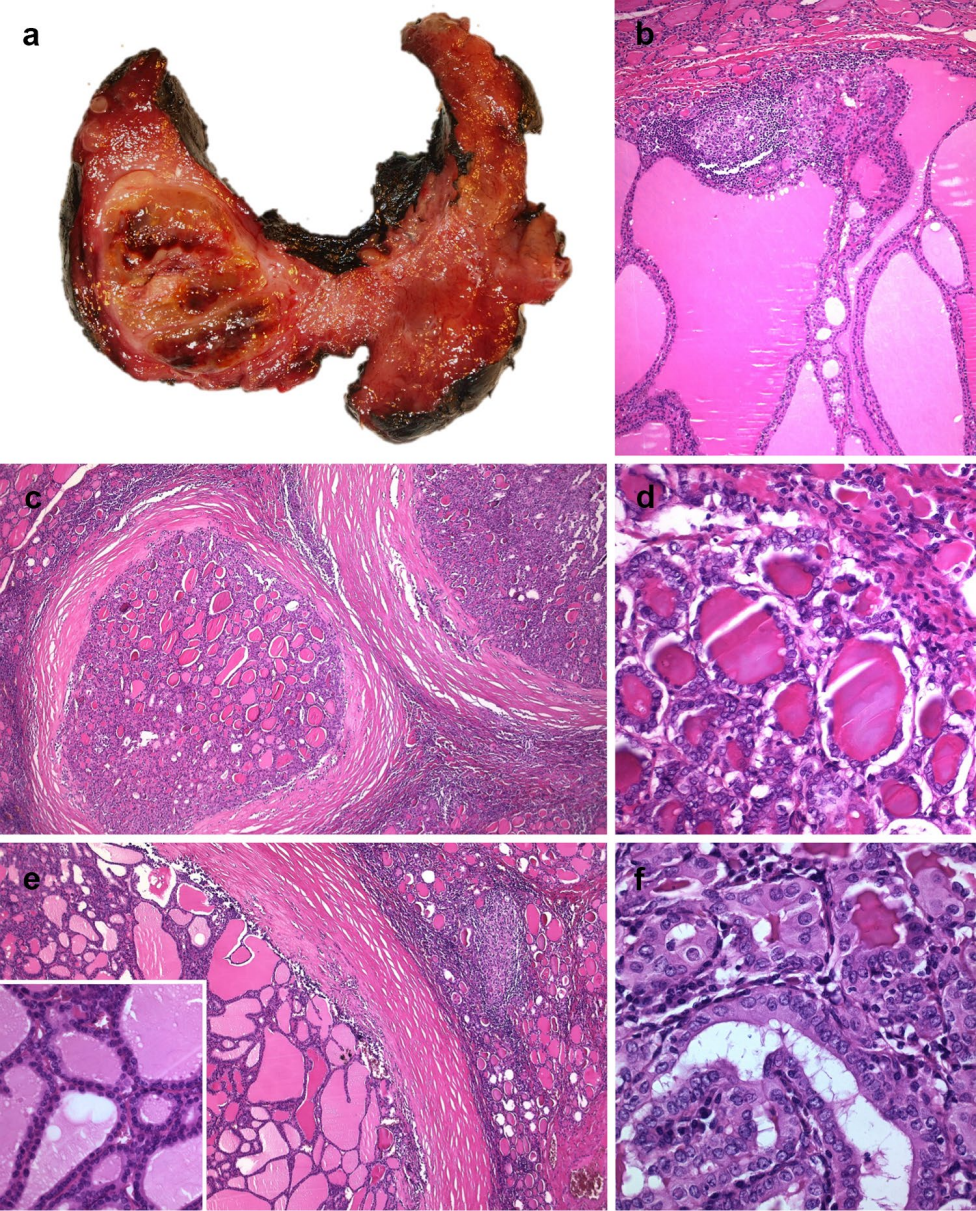
Non-syndromic familial non-medullary thyroid carcinoma case. Macroscopic appearance is not usually different from multinodular goiter (**a**). Histologically there is a combination of benign lesions (hyperplastic nodules as well as follicular adenomas) (**b**, **c**, **e**) with malignant follicular tumors. In this particular case, two papillary carcinomas can also be seen (**d**, **f**)

As previously mentioned, NSFNMTCs are primarily PTCs, both classical and follicular variants [10, 13, 19, 25, 28, 30–34], with more multifocality (with or without bilaterality) than in sporadic cases, usually in combination with benign lesions (hyperplastic nodules and/or follicular adenomas) [4, 10, 14, 22, 23, 26, 31–33, 35–39] (Fig. 1). Oncocytic change can also occur in these thyroid lesions [13, 40–43]. Synchronous PTC with follicular thyroid carcinoma (FTC), Hürthle cell carcinoma (HCC), and/or MTC have been occasionally reported [33].

Whether NSFNMTC is a more aggressive tumor remains a controversial issue. Compared with SNMTC, NSFNMTC is associated with a higher rate of lymph node metastases [3, 14, 28, 31, 38, 39], including lateral neck lymph node metastases [10] and Hashimoto’s thyroiditis [39]. NSFNMTC was also associated with extrathyroidal extension [14, 27, 38, 39] and recurrence [14, 44]. Aggressive features, however, were most apparent in certain families with three or more affected members [21, 23, 27, 45]. Nevertheless, several studies found no differences in the clinical behavior and outcome between sporadic and NSFNMTC [1, 2, 6, 14]. Therefore, a recent study concluded that although NSFNMTC is not more aggressive than SNMTC, this may not apply for the cases with three or more-affected relatives [21].

The usual coexistence of NSFNMTC with follicular nodular disease (including follicular epithelial cell hyperplasia to follicular adenoma progression sequence) suggests a multi-step process of tumor progression [23, 36], which together with its frequent multifocality justifies total thyroidectomy as the treatment for cases of NSFNMTC [10, 25, 26, 41]. Consistent with the current understanding of dynamic risk stratification in thyroid cancer, some studies indicate that familial papillary thyroid microcarcinoma is less aggressive than PTC greater than 1 cm and that a less invasive surgical treatment could be considered [46].

### Genetic Features

NSFNMTC is genetically heterogeneous and poorly understood [13, 32, 47–50]. A recent whole-genome sequencing of NSFNMTC identified germline alterations that highlighted the central role of PI3K/AKT and MAPK/ERK signaling pathways in this type of thyroid cancer [51]. The reported genes and loci associated with NSFNMTC are summarized in Table 1.

**Table 1:** Non-syndromic familial non-medullary thyroid carcinoma

Chromosomal loci (*designation*)	Gene	Thyroid lesions	Somatic thyroid tumor mutations	Additional lesions
9q22.23 [52–54]	*FOXE1*	PTC, FTC	*BRAF*^V600E^ [54]	
14q13.3 [55, 56]	*NKX2-1*	PTC, MNG		
12q14.2 [57]	*SRGAP1*	PTC		
15q23 [58]	*MAP2K5*	PTC		
20p12.3 [59–61]	*PLCB1*	PTC (follicular variant), MNG (papilloid adenomata)		
10q25.3 [62, 63]	*HABP2*	PTC, FTA		
1q41 [64]	*BROX*	PTC (classical and follicular variant)		
7q31.33 [65–68]	*POT1*	PTC (classical and follicular variant), HCC, HCA, MNG		Melanoma, dysplastic nevi
19q13.33 [69]	*NOP53*	PTC, HCC		
22q12.1 [70]	*CHEK2*	PTC	*BRAF*^V600E^ [70, 71]	Breast cancer [72]
19p13.11 [43]	*NDUFA13*	PTC (Hürthle cell variant), multiple Hürthle cell nodules		
19p13.2 [73]	*TIMM44*	PTC (classical and Hürthle cell variant), HCC, FTA with variable cell oxyphilia, multiple Hürthle cell nodules		
4q21.21 [74]	*ANXA3*	PTC (classical and follicular variant), HCC		
12q22 [74]	*NTN4*	PTC (classical and follicular variant), HCC		
14q32.13 [74]	*SERPINA1*	PTC		
17q21.2 [74]	*FKBP10*	PTC (classical and follicular variant)		
1p36.31 [74]	*PLEKHG5*	PTC (classical and follicular variant)		
17p13.2 [74]	*P2RX5*	PTC (classical and follicular variant)		
6p21.33 [74]	*SAPCD1*	PTC (classical and follicular variant)		
19p13.2 (*TCO/TCO1*) [13, 40–42, 75]	Unknown	PTC (classical and Hürthle cell variant), HCC, FTA with variable cell oxyphilia, multiple Hürthle cell nodules	19p13.2 LOH [76]	
8q24 (*PTCSC1*) [77]	Unknown	PTC, MNG, AITD		Melanoma
6q22 [33]	Unknown	PTC(classical and follicular variant), FTC#, HCC#, MTC#		
1q21 (*fPTC/PRN*) [33, 78]	Unknown	PTC, MNG		Papillary renal neoplasia [78]
14q32 (*MNG1*) [79]$	Unknown	PTC, MNG		
2q21 (*NMTC1*) [42, 75, 80]	Unknown	PTC (follicular variant)	2q21 LOH [75]	
8p23.1-p22 (*FTEN*) [81]	Unknown	PTC (classical and follicular variant), FTA, MNG	*BRAF*^V600E^ [81]	

*PTC* papillary thyroid carcinoma, *FTC* follicular thyroid carcinoma, *HCC* Hürthle cell carcinoma, *HCA* Hürthle cell adenoma, *FTA* follicular thyroid adenoma, *MNG* multinodular goiter, *AITD* autoimmune thyroid disease (Graves disease and Hashimoto’s thyroiditis)

^#^Concurrent FTC, HCC, and medullary thyroid carcinoma (MTC) was found in 3 classical PTC cases

^$^At least some cases are probably secondary to germline *DICER1* gene mutation

Although some studies support the involvement of germline *FOXE1/TTF-2* (9q22.23) variants [52–54, 82], this association is not entirely consistent [83–85]. Nuclear FOXE1 immunoexpression in tumor cells in the vicinity of the PTC border is associated with the presence of a risk allele of rs1867277 (c.-238G>A) in the 5′ untranslated region of the *FOXE1* gene, as well as with pathological characteristics (multifocality and capsular invasion) of PTC, suggesting possible FOXE1 involvement in the facilitation of tumor development [86].

Both *NKX2-1/TTF-1* (14q13.3) and *FOXE1* have been associated with the increased risk of sporadic PTC in Japan [82]. The *A339V NKX2-1* mutation may be a susceptibility gene for MNG and PTC [55], but this association could not be replicated in another NSFNMTC study [87]. Although association between the 14q13 locus and a predisposition to a Chinese familial form of MNG with PTC have been reported in a more recent study [56], further validation studies are required to demonstrate the clinical usefulness of testing this gene mutation in NSFNMTC cases.

*SRGAP1* (12q14.2) gene has been identified as a susceptibility gene in families with PTC [57]. The Q149H and R617C variants in *SRGAP1* could lead to a loss of function of the small G-protein CDC42 [57].

Recurrent genetic mutation of *MAP2K5* (15q23) variants c.G961A and c.T1100C (p.A321T and p.M367T) have been identified as susceptibility loci for NSFNMTC in Chinese families with PTC [58]; these mutations may result in an alternative activation of MEK5-ERK5 pathway in the MAPK signaling pathway.

The intronic *PLCB1* InDel is the first variant found in familial multiple papilloid adenomata-type MNG patients with more likelihood of progression to PTC and also found in a subset of patients with sporadic MNG [59, 60]. The InDel may contribute to MNG development through overexpression of phospholipase C beta 1 (PLCB1) (20p12.3) [60]. In this familial MNG of adolescent onset, the enlarged thyroid gland showed multiple nodules in a thyroid background of normal appearance. The nodules are sharply demarcated from the normal thyroid and are formed by follicles lined by follicular cells with small round regular nuclei and micropapillary projections [59–61]. This condition is different from the common MNG where the ill-defined nodules show large colloid rich follicles, and the background thyroid shows similar but less marked changes [61].

The pathogenic *HABP2* G534E variant has been associated with NSFNMTC [62, 63]. In addition, increased HABP2 protein expression in tumor samples from affected family members when compared with normal adjacent thyroid tissue and samples from sporadic cancers has been confirmed. [62]. Functional studies have shown that HABP2 has a tumor suppressive effect, whereas the G534E variant results in loss of function [62]. However, the role of the *HABP2* (10q25.3) gene in NSFNMTC is controversial [88–90] because the pathogenicity of *HABP2* variants in NSFNMTC could not be confirmed in Chinese, Brazilian and European studies [90–100]. Neither does this variant appear to play a role in sporadic PTC [101].

A new loss-of-function variant in *BROX* gene at 1q41 has been associated with the development of familial PTC (classical and follicular variants) [64], but more studies are needed to confirm this association. According to these researchers, *BROX* haploinsufficiency would induce altered EGFR degradation pathway in follicular cells, with EGFR accumulation and aberrant cell growth. [64].

*POT1* (protection of telomeres 1) gene is located at 7q31.33. Germline mutation in *POT1* has been reported in a melanoma-prone family with thyroid cancer and MNG [65, 66], as well as in a family affected solely by NSFNMTC [68]. Thyroid lesions included PTC, benign and malignant Hürthle cell neoplasms and MNG [65, 66, 68]. Loss-of-function or reduced activity of *POT1* seems to play a pathogenetic role via dysregulation of telomere protection [68]. However, a lack of mutations in the *POT1* gene in selected families with NSFNMTC, with at least three affected members, has been reported in another recent study [102].

*NOP53* gene, located in 19q13.33, encodes a nucleolar protein involved in ribosome biogenesis. The germline variant p. Asp31His in *NOP53* gene has recently been reported associated with NSFNMTC [69]. The patients had PTC and Hürthle cell carcinoma in one case, sometimes coexisting with MNG, including toxic MNG in two of the 11 affected members of the three families studied. Tumor tissue showed a higher immunohistochemical expression of NOP53 compared to the adjacent normal thyroid tissue in all four cases studied. [69].

*CHEK2* variants may be associated with NSFNMTC [70, 72, 103]. *CHEK2* (22q12.1) gene mutations may contribute to tumorigenesis through the haploinsufficiency mechanism due to low CHEK2 protein levels [70]. In fact, a lower intensity of nuclear immunostaining for CHK2 protein has been detected in PTC cases with the *CHEK2* Y139X variant than in sporadic PTC cases [70]. A germline *CHEK2* mutation has been found in seven of 11 women (63%) with multiple primary cancers of the breast and thyroid [72]. Rare missense variants (R180C and H371Y) in cell cycle checkpoint kinase 2 (*CHEK2*) have also been identified in 2% of patients in a series of sporadic PTC [70]. Coexistence of *CHEK2* and *BRAF*^V600E^ mutations has been reported in 10.8% of 427 unselected PTC patients, including mainly cases of the classical variant, but also of the follicular, oxyphilic, diffuse sclerosing, and solid variants [71]. In the same series, the coexistence of both mutations, however, was not associated with more aggressive clinicopathological features of PTC, poorer treatment response, or disease outcome.

Benign and malignant thyroid tumors with an oncocytic phenotype have been associated with germline [43] and somatic mutations [43] in the *NDUFA13/GRIM-19* (19p13.11) gene. Another group of familial oxyphilic (oncocytic) thyroid tumors has been associated with the *TCO* (thyroid tumors with cell oxyphilia) gene, which has been mapped to chromosome 19p13.2 [13, 40–42, 75]. Interestingly, a systematic screening of candidate genes mapping to the region of linkage in affected *TCO* members, led to the identification of novel germine changes in the *TIMM44* (19p13.2) gene [73]. In the same chromosomal region (19p13.2), a germline *KEAP1* gene mutation has been reported in a Japanese family with MNG but not thyroid cancer [104].

Mainly based on linkage analysis several susceptibility loci for NSFNMTC have been also proposed (Table 1).

A *PTCSC1* (papillary thyroid carcinoma susceptibility candidate 1) gene in 8q24 as a candidate gene for PTC predisposition was identified through linkage, haplotype sharing, and gene expression analysis in families with PTC, MNG, autoimmune thyroid disease (Graves’ disease and Hashimoto’s thyroiditis), melanoma and other malignancies [77]. This association between 8q24 and risk for thyroid cancer and other cancers (prostate cancer, colorectal cancer, breast cancer, etc.), has been confirmed in recent studies [105, 106].

Linkage was identified with 2 single-nucleotide polymorphism markers on chromosomal loci 6q22 and 1q21 in families with classical and follicular variant of PTC (in some cases coexisting with FTC, HCC, or MTC) and FTC [33]. Interestingly, *FPTC/PRN* (familial PTC/papillary renal neoplasia) gene has also been mapped to 1q21 region in families with PTC, MNG, as well as, benign and malignant renal papillary neoplasms [33, 78].

Locus 14q (*MNG1* (multinodular goiter 1)) has been described as a susceptibility gene in families with adolescent-onset goiter, PTC and FTC [79]. Based on the chromosomal location and the type of lesions (multinodular goiter in childhood, beningn thyroid tumors, differentiated thyroid cancer, rhabdomyosarcoma, as well as ovarian and brain tumors) described in these families [79], we think that at least some cases are secondary to germline *DICER1* gene mutation [107] (see *DICER1* syndrome below). Loci Xp22 (*MNG2*) [108, 109] and 3q26.1-q26.3 (*MNG3*) [110] have been also associated with cases of familial non-toxic multinodular thyroid goiter but not with NSFNMTC.

A susceptibility locus 2q21 (*NMTC1* (non-medullary thyroid carcinoma 1) gene) has been found in a large Tasmanian family with PTC (follicular variant) and no cell oxyphilia or renal cancer [80]. There is also evidence that *NMTC1* (2q21) and *TCO* (19p13.2) may interact to increase risk in individuals that inherit both susceptibility genes [42, 75].

Another familial thyroid neoplasia susceptibility locus on 8p23.1-p22 called *FTEN* (familial thyroid epithelial neoplasia) has been described in a large Portuguese family with benign thyroid lesions and PTC [81].

An ultra-rare mutation (4q32A.C) involved in the predisposition to both PTC and ATC has been reported in a family with non-medullary thyroid cancer [111]. This mutation is located in a long-range enhancer element whose ability to bind the transcription factors *POU2F* and *YY1* is significantly impaired, with decreased activity in the presence of the C-allele compared with the wild type A-allele. An enhancer RNA is transcribed in thyroid tissue from this region and is greatly downregulated in NSFNMTC [111].

A recent study from Brazil also identified seven novel germline variants in familial PTC; these include p.D283N*ANXA3, p.Y157S*NTN4, p.G172W*SERPINA1, p.G188S*FKBP10, p.R937C*PLEKHG5, p.L32Q*P2RX5, and p.Q76*SAPCD1 [74].

The *BRAF*^V600E^ mutation is not a germline mutation in NSFNMTC [112]; however, *BRAF*^V600E^ [54, 70, 71, 81] as well as *HRAS* and *NRAS* [81] somatic mutations have been found in some cases of NSFNMTC, raising the possibility of mutations in DNA repair genes that prevent these sporadic mutations.

## Syndromic Familial Non-Medullary Thyroid Carcinoma (SFNMTC)

In this group, the FNMTC is associated with syndromes having extrathyroid manifestations [9, 49, 113–115] (Table 2). The well-defined familial syndromes that are closely linked to FNMTC include familial adenomatous polyposis (FAP) syndrome, PTEN-hamartoma tumor syndrome, DICER1 syndrome, Carney complex, and Werner syndrome; however, patients with MEN1 syndrome [118] Marfan syndrome [119] and familial paraganglioma syndromes caused by *SDHx* mutations [120] can also manifest with thyroid follicular epithelial-derived neoplasia. It is not clear if the association with papillary thyroid microcarcinomas in multiple endocrine neoplasia type 2A (MEN2A) patients is related to specific germline changes of the *RET* gene or is reflective of how carefully the thyroids of MEN2A patients are examined [121, 122]. Recognition of these syndromes is important so that cancer screening and genetic counseling can be initiated. Pathologists also play an important role in recognizing the syndromes addressed below [123–125]. Given the well established genotype–phenotype correlations, this section will focus on well-defined thyroid neoplasia-related familial syndromes.

**Table 2:** Syndromic familial non-medullary thyroid carcinoma

Syndrome (inheritance)	Gene (gene location)	Thyroid lesions (incidence)	Main additional lesions
Familial adenomatous polyposis (autosomal dominant)	*APC* (5q22.2)	Cribriform-morular variant of TC (16%)	Colorectal adenomatous polyps, colorectal adenocarcinoma, CHRPE, desmoid tumors (GAPPS)
PTEN-hamartoma tumor syndrome (autosomal dominant)	*PTEN* (10q23.31)	MNG (43–75%) FTA (25%), HCA, lipoadenoma, and microadenomas PTC (microcarcinoma, classical and follicular variant) (60%) FTC (14–45%) HCC (≈ 1%) ATC (< 1%) C-cell hyperplasia Lymphocytic thyroiditis (55%)	Adult Lhermitte-Duclos disease^P^*, mucocutaneous lesions (facial trichilemmomas^P^, papillomatous papules^P^, acral keratosis^P^), autism spectrum disorder^P^, breast cancer^M^, macrocephaly^M^, endometrial carcinoma^M^, mucocutaneous lesions (multiple palmoplantar keratosis^M^, multifocal cutaneous facial papules^M^, macular pigmentation of the glans penis^M^, multiple gastrointestinal (GI) hamartomas^M^ or ganglioneuromas^M^, FTA^m^, MNG^m^, single GI hamartoma^m^ or ganglioneuroma^m^, fibrocystic breast disease^m^, lipomas^m^, fibromas^m^, genitourinary tumors (particularly kidney carcinoma)^m^, genitourinary malformations^m^, uterine fibroids^m^, autism spectrum disorder^m^
DICER1 syndrome (autosomal dominant)	*DICER1* (14q32.13)	TC (PTC, FTC, PDTC) (rare)** FTA MNG	Pleuropulmonary blastoma, pulmonary cysts, cystic nephroma, Sertoli-Leydig cell tumor, gynandroblastoma, juvenile granulosa cell tumor, ciliary body medulloepithelioma, nasal chondromesenchymal hamartoma, embryonal rhabdomyosarcoma, pituitary blastoma, pineoblastoma, central nervous system sarcoma, presacral malignant teratoid tumor
Carney complex (autosomal dominant)	*PRKAR1A* (17q24.2)	TC (FTC, PTC) (15%) FTA MNG	Pigmentation in skin and mucosa (lips, conjunctiva and inner or outer canthi, penile and vaginal mucosa), multiple myxomas (cutaneous, mucous, cardiac and/or in the breast), primary pigmented nodular adrenocortical disease, large-cell calcifying Sertoli cell tumors, growth hormone-producing pituitary adenoma, blue nevus, epithelioid blue nevus, breast ductal adenoma, osteochondromyxoma
Werner syndrome (autosomal recesive)	*WRN* (8p12)	TC (18%) (FTC, PTC, ATC) FTA	Premature graying and/or thinning of scalp hair, bilateral ocular cataracts, deep, chronic ulcers around the ankles, short stature, melanoma, meningioma, soft-tissue sarcomas, leukemia and preleukemic disorders, osteosarcomas

*TC* thyroid cancer, *CHRPE* congenital hypertrophy of the retinal pigment epithelium, *GAPPS* gastric adenocarcinoma and proximal polyposis of the stomach, *MNG* multinodular goiter, *FTA* follicular thyroid adenoma, *HCA* Hürthle cell adenoma, *PTC* papillary thyroid carcinoma, *FTC* follicular thyroid carcinoma, *HCC* Hürthle cell carcinoma, *ATC* anaplastic carcinoma

^*^P, pathognomonic criteria; M, major criteria; m, minor criteria; according to the International Cowden Consortium operational diagnostic criteria [116]

^**^There is a 16- to 24-fold increased risk of TC [117]

### Familial Adenomatous Polyposis (FAP) Syndrome

FAP is an autosomal dominant syndrome caused by inactivating germline *APC* gene mutations and characterized by multiple colorectal adenomatous polyps and a high risk of colorectal, thyroid and other cancers [126]. *Classic FAP* is usually associated with the development of numerous (hundreds to thousands) colorectal adenomatous polyps and a nearly 100% risk of developing colorectal adenocarcinoma [127]. *Attenuated FAP* is characterized by fewer (20–100) adenomas in the large bowel, as well as both a slightly lower risk and later onset of colorectal cancer [128]. FAP also correlates with extracolonic lesions including congenital hypertrophy of the retinal pigment epithelium (CHRPE) [129], desmoid tumors [130], gastric adenocarcinoma and proximal polyposis of the stomach (GAPPS) [131], duodenal [132] and hepatobiliary tree tumors, hepatoblastoma, adrenocortical adenomas and carcinomas, osteomas, epidermal cysts, and extranumerary teeth [133]. *Gardner syndrome* is an obsolete term, and its use is not recommended since almost all FAP patients have these characteristics [126]. Although the association of FAP and brain tumors, commonly medulloblastomas, is referred to as *Turcot syndrome*, most cases are actually due to constitutional pathogenic mutations affecting the DNA mismatch repair genes, *MLH1*, *MSH2*, *MSH6*, and *PMS2* [126].

The thyroid tumor associated with FAP is the cribriform-morular variant of PTC, more recently designated as cribriform-morular variant of thyroid carcinoma [134, 135]. The prevalence of the cribriform-morular variant (C-MV) of TC among FAP patients reaches up to 16% when ultrasonographic screening is combined with fine needle aspiration biopsy (FNAB) [136], and this is more common in young women (mean age 26 years, range 8–61 years, with a ratio women/men of 61:1, respectively) [134, 137, 138]. In these families the diagnosis of C-MV of TC precedes that of FAP in up to 40% of the cases [134]. Patients are generally euthyroid and C-MV of TC is sonographically more like a follicular tumor or MNG rather than PTC [134, 137]. Compared with sporadic cases, C-MV of TC associated with FAP are usually multifocal (and bilateral) tumors [134, 137].

#### Pathological Features

Although to the best of our knowledge, Crail reported the first case of C-MV of TC in a patient with FAP [139], it was Harach HR et al. who highlighted the peculiar microscopic characteristics of FAP-associated follicular cell-derived thyroid carcinoma, which usually presents as a multifocal and/or bilateral tumor [140]. Because of its distinctive histological features, Cameselle-Teijeiro and Chan proposed the term “cribriform-morular variant of PTC” and reported apparently-sporadic tumors which, unlike the syndromic type, usually appear as a single nodule [141].

**Fig. 2:**
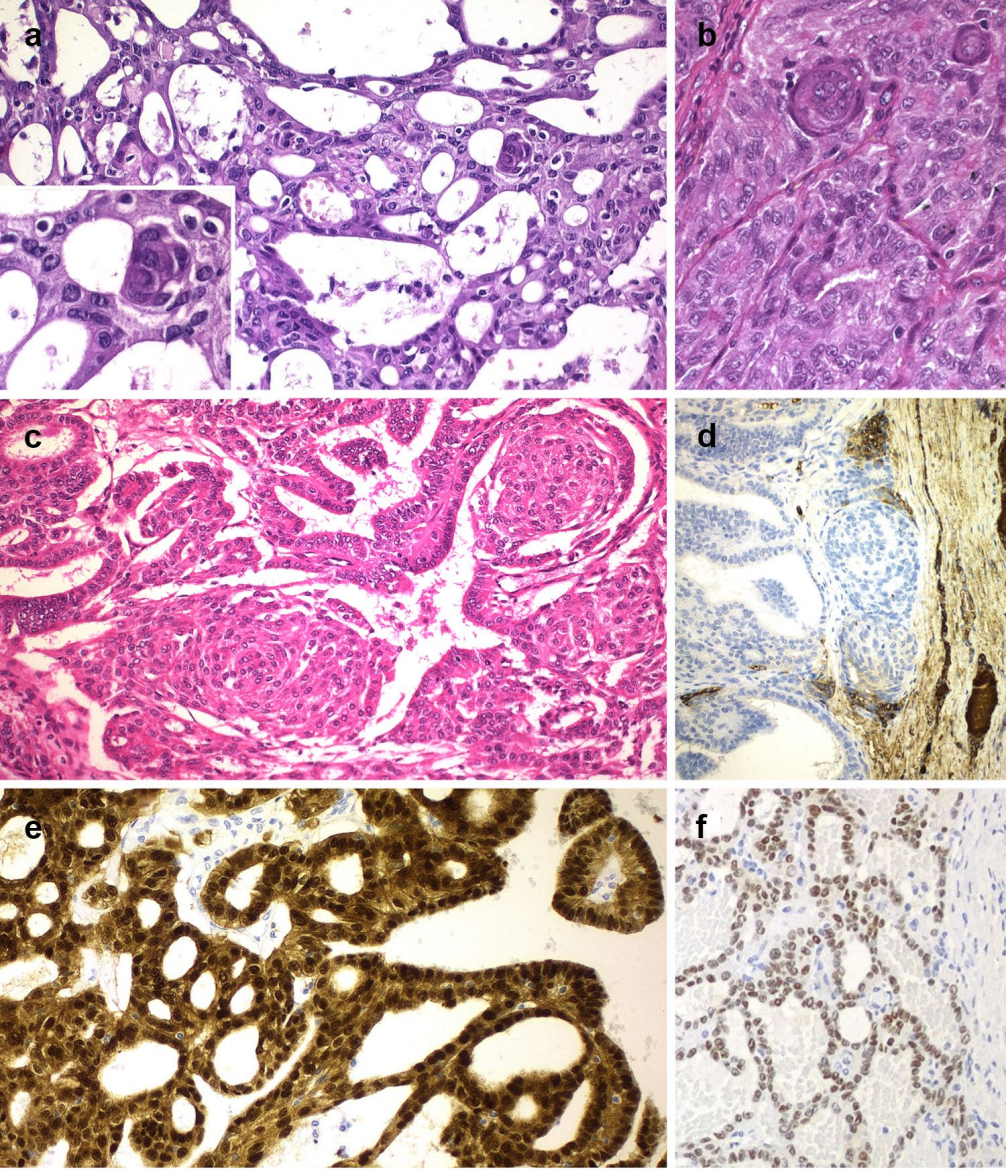
Cribriform-morular variant of thyroid carcinoma. This tumor is usually encapsulated or well-defined, partially divided by fibrous septa. Histologically, there is a mixture of cribriform (**a**), papillary (**c**), follicular, trabecular (**b**) or morular (squamoid) (**a**–**c**) patterns. Papillary or pseudopapillary structures are lined by cuboidal or columnar cells (**c**). Follicular and cribriform areas are usually devoid of colloid (**a**, **c**, **d**). Tumor cells are usually negative for thyroglobulin; focal positivity may represent trapped nontumorous tissue (**d**). Tumor cells are always positive for TTF1 (**f**). Nuclear and cytoplasmic positivity for β-catenin is the hallmark of the cribriform-morular variant of thyroid carcinoma (**e**)

C-MV of TC usually presents as a solid, white, fleshy, encapsulated, or well-defined nodule, partially divided into lobes by fibrous septa [134, 140, 141]. Histologically, there is a mixture of papillary, follicular, cribriform, trabecular, and morular (squamoid) growth patterns (Fig. 2a-f) Pseudopapillary and non-branched papillary structures are lined by cuboidal or columnar cells. The tumors, including areas of follicular and cribriform patterns, are usually devoid of colloid. In the solid areas there are oval to plump spindle cells and morules with aggregates of biotin-rich nuclei with a peculiar chromatin clearing. The tumor cells have abundant eosinophilic cytoplasm and the nuclei are usually hyperchromatic with a variable presence of nuclear features of PTC such as pallor, nuclear grooves, intranuclear cytoplasmic inclusions, and overlapping. The mitotic activity is generally less than 5 per 10 high power fields. Necrosis is uncommon. Capsular invasion and vascular invasion have been reported in about 40% and 30% of cases, respectively. Psammoma bodies are not frequent [134]. Although the cribriform pattern of growth formed by anastomosing arches of tumor cells with non-fibrovascular stroma and morules are characteristic of C-MV of TC, a variable proportion of different patterns of growth can be found even among different tumors of the same patient. Adenoid cystic carcinoma-like areas have been reported in one case [142]. C-MV of TC with poorly differentiated features has also been reported [143].

Tumor cells are negative or focally positive for thyroglobulin (Fig. 2d) but always positive for TTF1 (Fig. 2f), and always negative for calcitonin and cytokeratin 20. Nuclear and cytoplasmic positivity for β-catenin is the hallmark of the C-MV of TC, in contrast to the membranous pattern in normal follicular cells. This is the only thyroid tumor with both nuclear and cytoplasmic positivity for β-catenin [134] (Fig. 2e). The role of LEF-1 as a marker of this TC still needs confirmation [144]. Tumor cells are also immunoreactive for keratins using the pankeratin clone AE1/AE3, low molecular weight keratin clone CAM5.2, and clone 34βE12 that identifies cytokeratins 1, 5, 10, and 14; they express epithelial membrane antigen, E-cadherin, vimentin, galectin-3, BCL-2, and p27 and are stained by the Hector Battifora mesothelial cell-1 (HBME1) antibody. Strong positivity for progesterone receptors and for α and β-estrogen receptors, as well as focal positivity for androgen receptors is usually detected [134]. Morulae are distinct from squamous metaplasia; the morules show nuclear positivity for β-catenin, are selectively positive for CDX2, CA19.9, and CD10, and are negative for TTF1, thyroglobulin, calcitonin, vimentin, and BCL2 [134, 145, 146].

Because of the morphological features of C-MV of TC, distinction from other primary or metastatic lesions may be necessary [147]. C-MV of TC can simulate a colonic or metastatic breast carcinoma, but positivity for TTF1 can help determine the correct diagnosis. The lack of morular structures, positivity for thyroglobulin and negativity for nuclear β-catenin distinguish columnar cell variant of PTC from C-MV of TC. Solid areas in the C-MV of TC can simulate a poorly differentiated thyroid carcinoma, but coexistence with a cribriform and/or morular pattern together with a lower mitotic index are typical of the C-MV of TC [147]. On the other hand, lung metastasis from C-MV of TC should not be misinterpreted as a primary adenocarcinoma of the lung based exclusively on the positivity for CK7 and TTF1 [148] can be helpful in this situation.

FNAB samples from C-MV of TC are commonly indicative of thyroid carcinoma, showing hypercellularity, papillary structures, epithelial flat monolayers, and/or morular structures [134, 137, 146, 147]. Tumor cells are tall and columnar with spindle cytoplasm and obscure nuclei, but typical nuclear features of PTC (grooves, pallor, and cytoplasmic inclusions) are usually seen. The presence of cribriform and/or morular areas as well as nuclear staining for β-catenin however, are the keys to the diagnosis of C-MV of TC [134, 147].

C-MV of TC generally has a good clinical course. Extrathyroidal extension, local recurrence, lymph node metastases, distant metastases, and death were reported in 4%, 4.5%, 10%, 6%, and 3% of cases taken from a review of 134 cases of C-MV of TC [134, 149]. Tumors with poor differentiation [143], neuroendocrine differentiation [150], and/or *TERT* promoter mutations [151] have been associated with a worse prognosis. High Ki-67 labeling indexes (22–70%) have been reported in cases of C-MV of TC with pulmonary metastases [152, 153], one of them having a good response to treatment with lenvatinib [153]. However, Ki-67 not associated with numerous mitoses does not seem to portend greater biological aggressiveness [154].

#### Genetic Features

FAP is caused by germline (constitutional) mutations in the *APC* gene (5q22.2) that result in a truncated or absent APC protein [126]. The severity of FAP changes according to the site of the germline *APC* gene mutation. In classic FAP, disease-associated germline mutations tend to be clustered in a small region designated the mutation cluster region (MCR), around codon/amino acid 1309 (codons 1286 to 1513) [132, 155]. In attenuated FAP, associated inherited mutations are located nearer the N-terminus or within the alternatively spliced section of exon 9 [128]. In more than 80% of patients with FAP and thyroid cancer, *APC* germline gene mutations occur between codons 140 and 1513 (largely outside the MCR) [137, 138, 156, 157]. Most *APC* germline mutations associated with thyroid cancer occur in the 5′-portion of exon 15, in the same genomic area associated with CHRPE (codons 463–1387), and codon 1061 is also a hot spot for both C-MV of TC and hepatoblastoma [158]. While the risk of hepatoblastoma is greater in children younger than 3 years, the risk of developing thyroid cancer is greatest during the second and third decade of life [159]. For this reason, the fundoscopic confirmation of CHPRE could be an indicator of the familial character of a case of C-MV of TC while awaiting the result of the definitive genetic studies [129].

In normal follicular epithelial cells, APC protein forms a destruction complex with glycogen synthetase 3β (GSK3), casein kinase 1α and axin 1, sequestering β-catenin, and targeting it for degradation [160]. When the WNT pathway is activated, the destruction complex is uncoupled so the unphosphorylated β-catenin accumulates in the cytoplasm, translocates to the nucleus and activates transcription factors involved in proliferation and loss of differentiation. In multicentric thyroid tumors of FAP patients, it has been found that each tumor has a different somatic *APC* mutation (second hit), suggesting an independent development of each tumor through biallelic inactivation of the *APC* gene [157, 161]. The relationship between the morphology and the accumulation of beta-catenin in the C-MV of TC and the *APC* gene has also been confirmed in sporadic cases in which a somatic *APC* mutation in exon 15 at codon 1309 has been detected, with a dominant negative effect [162]. Two somatic (biallelic), inactivating *APC* variants have been identified in a sporadic case of C-MV of TC [163]. There are also sporadic cases of C-MV of TC associated with missense somatic mutations of exon 3 of the β-catenin gene (*CTNNB1*) without mutations or loss of heterozygosity (LOH) of the *APC* gene [164]. More recently, *AXIN1* somatic mutations (exons 1 and 7) have also been reported in a sporadic and a familial case of C-MV of TC, respectively [165, 166]. The set of these molecular alterations underscores the key role of the WNT/β-catenin signaling pathway in the development of both sporadic and familial forms of C-MV of TC.

Somatic molecular alterations typical of conventional (not hereditary) thyroid cancer such as *RET/PTC* rearrangements have been reported in some familial cases of C-MV of TC [150, 167–169]. *KRAS*, but not *HRAS* nor *NRAS* has been detected in 7.6% of these tumors, including in one sporadic case [170] and in another FAP-associated case [165]. *PIK3CA* somatic mutations (exon 9, codon 545) have been reported in 3 sporadic cases of C-MV of TC [171]. *TERT* promotor mutation has been reported in a sporadic case with aggressive behavior [151]. Rare somatic mutations in thyroid tumors such as in the *KMT2C* and *KMT2D* genes have been described in 1 and 4 cases of C-MV of TC, respectively, coexisting with the germline mutation of the *APC* gene [138]. In this same series of patients with FAP and thyroid tumors, *BRAF*^V600E^ somatic mutations have been detected in conventional PTCs but not in the cases displaying a C-MV phenotype [138]. Neither *BRAF*^V600E^ mutations nor *PAX8/PPARγ* rearrangements have been described in the C-MV of TC [138, 151, 165, 170–172].

It has been proposed that the sporadic cases of C-MV of TC result from a combination of somatic mutations in phenotypically equivalent genes such as *APC*, *CTNNB1*, and/or *AXIN1* [134]. A similar possible pathogenetic mechanism has also been proposed for somatic mutations in the *KMT2D* gene. [136]. Somatic mutations in *RAS*, *PIK3CA*, *KMT2C*, and/or *BRAF*^V600E^, as well as somatic *RET/PTC* rearrangements could act as a somatic “second-hit” in the development of other forms of thyroid cancers in affected individuals [9, 134, 138]. A tumor growth-promoting role has been attributed to the sex hormones due to the striking predominance of C-MV of TC in women and the strong expression of estrogen and progesterone receptors by tumor cells. [134]. Sporadic thyroid carcinomas unrelated to the pathogenesis of C-MV of TC may also occur in the thyroid of patients with FAP.

### PTEN Hamartoma Tumor Syndromes (PTHS)

PTEN hamartoma tumor syndrome (PHTS) is an autosomal dominant disorder caused by inactivating germline *PTEN* gene mutations [173–176]. PHTS patients have diverse phenotypes such as *Cowden disease* (CS), *Bannayan-Riley-Ruvalcaba syndrome* (BRRS), *PTEN-related Proteus syndrome* (PS), and *Proteus-like syndromes*, but the PHTS designation should be used for all these syndromes, when a germline *PTEN* mutation is detected [173, 175, 177]. CS is a multiple hamartoma syndrome presenting in adulthood usually having macrocephaly, a high risk for benign and malignant tumors of the thyroid, breast and endometrium, multiple hamartomas and also various other lesions as well (see Table 2). BRRS (including *Bannayan-Rubalcaba-Riley syndrome*, *Bannayan-Zonana syndrome*, and *Myhre-Riley-Smith syndrome*) is a pediatric syndrome typically displaying macrocephalia, intestinal hamartomatous polyposis, lipomas, penile freckling, and the same cancer risk as CS [173, 174, 176, 177]. PS and Proteus-like syndromes are congenital disorders associated with malformations and hamartomatous tissue overgrowths, connective tissue nevi, hyperostoses and other lesions [173–176]. Most of these disorganized overgrowths of essentially mesenchymal elements have been more recently termed PTEN hamartoma of soft tissue (PHOST) [178]. In rare cases of Cowden and Cowden-like syndromes as well as in proteus syndrome, other susceptibility genes have been reported (see below) [179–182].

The risk of TC in patients with PHTS ranges from 14 to 38% [183–187]. TC can occur at any age although it is more common in young adults (median 44 years), predominates in women and its clinical features are similar to those of sporadic TC [180, 187, 188]. Because thyroid cancer can occur in early childhood, ultrasound surveillance for all patients with pathogenic germline *PTEN* variants, regardless of their age, has been proposed [189]. Due to the fact that thyroid tumors are multifocal and there is increased risk of early progression to thyroid carcinoma, total thyroidectomy instead of lobectomy has been recommended in these patients [123, 185, 190].

#### Pathological Features

**Fig. 3:**
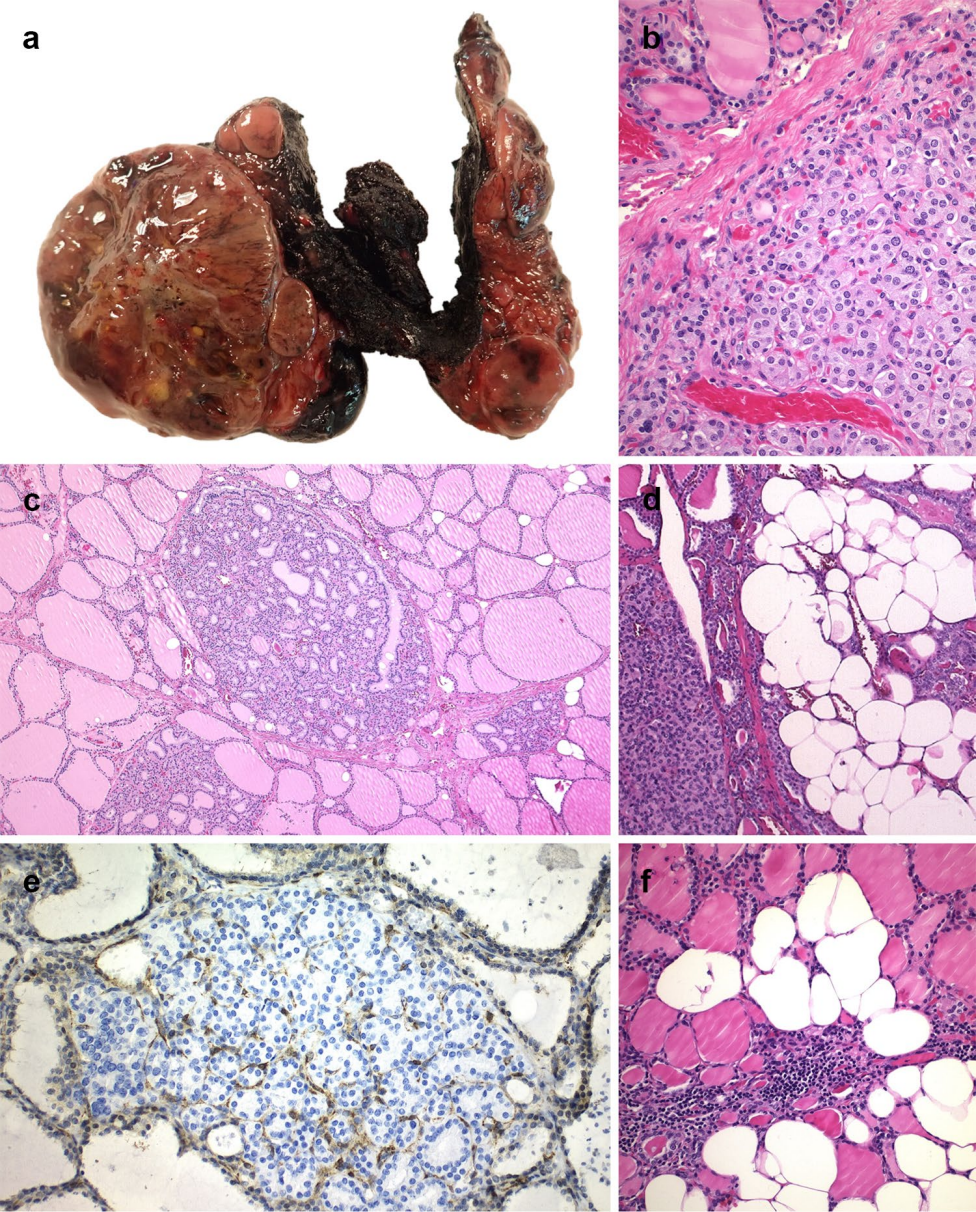
PTEN-hamartoma tumor syndrome (PHTS). In this case, thyroid involvement is typically multinodular and bilateral (**a**). Microscopically, the presence of multiple bilateral adenomatous nodules (“microadenomas”) (**c**) and adenomas (**b**), including adenolipomas (**d**), is characteristic. Adipocytic infiltration and lymphocytic thyroiditis can also be seen (**f**). The loss of PTEN protein expression in thyroid nodules, whether in all nodules or in a subset of nodules with expression in endothelial cells (internal positive control), is both sensitive and specific for PHTS (**e**)

As appears in the first description of Cowden syndrome in 1963 [191], thyroid involvement is typically multinodular and bilateral, with no pathological differences between the thyroid findings in CS [123, 125, 187, 192] and BRRS [189, 192] (Fig. 3a). The thyroid gland in PHTS shows multifocal adenoma-to-carcinoma progression sequence. Histologically, follicular epithelial-derived thyroid carcinomas arise in the background of multiple cellular follicular adenomas (Fig. 3b) including lipoadenomas (Fig. 3d) as well as numerous cellular nodules (so-called microadenomas) [123, 125, 188, 189] (Fig. 3c, e).

Multinodular goiter due to multiple follicular adenomas appears in 43–75% of patients, sometimes including oncocytic or clear cells or a hyalinizing trabecular tumor-like pattern [123, 187, 192, 193]. The most common types of malignant tumors are PTC (60%), including the follicular variant of PTC, FTC (14–45%), poorly differentiated and anaplastic thyroid carcinoma (ATC) (6%) [123, 185, 187, 192]. Although reactive (secondary) C-cell hyperplasia has been reported in about 55% of cases [194], MTC is not a component of PHTS; scattered foci of adipose tissue distributed throughout the thyroid parenchyma as well as thyroiditis (75%) are also characteristic [116, 125, 175, 187, 192, 195] (Fig. 3f).

It is very important for pathologists to know that immunohistochemical staining of thyroidectomy specimens for PTEN protein can aid in the identification of patients with PHTS. By immunohistochemistry, the loss of PTEN protein expression in all follicular-epithelial derived thyroid nodules (including microadenomas), whether in all nodules or in a subset of nodules, is both sensitive (100%) and specific (92.3%) for CS [188]. In fact, in PHTS cases there is a loss of PTEN expression in neoplastic follicular cells, while the normal follicular cells and endothelial cells (positive control) are positive [9, 114, 125, 147, 188, 196] (Fig. 3e).

#### Genetic Features

PHTS is caused by pathogenic germline *PTEN* variants (10q23.31). The PTEN protein encoded by this tumor suppresor gene is a phosphatidylinositol-3,4,5-trisphosphate 3-phosphatase that canonically counteracts the PI3K/AKT/mTOR signaling pathway [173]. Mutation or inactivation of the *PTEN* gene increases PIP3 levels causing constitutive activation of AKT with subsequent upregulation of mTOR signaling; this implies more cell proliferation, migration, angiogenesis, survival and decreased apoptosis [175, 197]. PTEN also exerts protein phosphatase-dependent and pan-phosphatase-independent actions within both the cytoplasm and the nucleus [175]. *PTEN* mutations could affect the amount of protein, causing haploinsufficiency, acting as dominant-negative, reducing or losing phosphatase activity, and/or producing abnormal localization and function [198]. In PHTS, germline mutations can affect all nine exons of the *PTEN* gene, with approximately two thirds of mutations occurring in exons 5, 7, and 8 [116, 192]. Up to 40% of all these mutations appear in exon 5, encoding the core catalytic motif [116, 175]. In addition to intragenic mutations, germline *PTEN* promoter mutations have been described in about 10% of PHTS patients [199, 200]. Furthermore, large *PTEN* deletions in about 3–10% of these patients have also been found [116, 175, 199]. Germline *PTEN* frameshift mutations have been reported to be overrepresented in TC [180], but no correlation has been found between specific germline mutations and thyroid pathological features [192].

Interestingly, in about 6% of PHTS patients, some germline variants in genes that encode subunits of mitochondrial complex II such as *SDHB*, *SDHC*, and *SDHD* (*SDHx*) can act as modifiers of PTEN-associated cancer risk and tumor histology [201]. In fact, individuals carrying the *SDHx* variants showed an increased risk of PTC, breast cancer, and renal cell cancer that exceeds the risk mediated by mutant *PTEN* alone [201]. In patients with only *PTEN* mutation, the risk of breast cancer was 32.4% and TC 25.7%, mainly FTC. With only *SDHx* variants, there was an increase in breast cancer (57.4%) and TC (51.1%) but with a predominance of PTC (including the follicular variant of PTC). But with the mutation of both *PTEN* and *SDHx* genes, the risk of breast cancer was greater (77.2%), the risk of TC decreased (27.3%), and the risk of renal cancer disappeared [201, 202].

In some studies [181], pathogenic germline *PTEN* variants have only been detected in about 25% of CS/CS-like individuals meeting the International Cowden Consortium criteria [116], and pathogenic germline *PTEN* variants have also been found in up to 11% of BRRS patients [199]. Thus, it has been postulated that in these individuals with no germline *PTEN* variants, germline mutations in other genes with a similar functional effect to those of PTEN malfunction, would explain the equivalent phenotype [202]; in fact, pathogenic germline variants in *PIK3CA* and *AKT1* [181] as well as in *EGFR* [203] genes have been reported in CS and CS-like individuals. Mutations in non-PTEN pathway genes associated with CS and BRRS that could explain the remaining patients are *SDHB*, *SDHC*, *SDHD*, *KLLN* (epimutation), *SEC23B*, *USF3*, *TTN*, *PTK2*, and *RASAL1* [179, 180, 202, 204].

A large deletion in the mitochondrial-DNA-encoded *MT-ND1* and a somatic *BRAF*^V600E^ mutation have been reported in a HCC and in a PTC respectively in the thyroid gland of a CS patient [205]. No *BRAF*^V600E^, *NRAS* or *KRAS* somatic mutations were detected in one PTC (follicular variant), one FA, two adenolipomas and two adenomas of another patient with CS [125].

### *DICER1* Syndrome

*DICER1* syndrome is an autosomal dominant disorder with decreased penetrance, caused by heterozygous inactivating germline *DICER1* gene mutations. *DICER1* syndrome, also designated as *Pleuropulmonary blastoma familial tumor* and *Dysplasia syndrome*, is characterized by an increased risk for pleuropulmonary blastoma, pulmonary cysts, cystic nephroma, benign and malignant thyroid tumors, Sertoli-Leydig cell tumor, gynandroblastoma, juvenile granulosa cell tumor, ciliary body medulloepithelioma, nasal chondromesenchymal hamartoma, embryonal rhabdomyosarcoma, pituitary blastoma, pineoblastoma, central nervous system sarcoma, and presacral malignant teratoid tumor [206–209] (see Table 2). Additional lesions include macrocephaly, ocular and dental abnormalities, and structural alterations of the kidney and collecting system [209]. Due to its rarity, the diagnosis of pleuropulmonary blastoma, cystic nephroma or nasal chondromesenchymal hamartoma, is highly suggestive of germline *DICER1* mutation [206, 207]. Pituitary blastoma, appears to be pathognomonic of biallelic *DICER1* mutations [210, 211]. All these lesions usually occur in childhood, adolescence, or early adulthood [206–209].

An epidemiological study of individuals with one or more *DICER1*-associated lesions showed that by 20 years of age, the cumulative incidence of multinodular goiter or history of thyroidectomy is 13% in men and 32% in women, with a 16- to 24-fold increased risk of TC over a patient’s lifetime [117]. Early-onset, familial, or male MNG should also alert to the possibility of *DICER1* syndrome, especially in the case of a family history of other *DICER1*-associated cancers [117, 212]. In early onset of MNG, the head circumference should also be measured, because it is also associated with *DICER1* syndrome [117], but in the absence of germline *DICER1* mutation, PHTS should also be considered (see above). For individuals with a *DICER1* pathogenic variant, thyroid ultrasound is recommended beginning at age 8 with subsequent ultrasounds every 3 to 5 years [206]. Although cases of TC reported in children had been attributed to the chemotherapy and/or radiation they had received for *DICER1*-associated tumors [213–215], germline *DICER1* mutations are associated with an increased risk of developing familial differentiated TC, even in the absence of prior treatment with chemotherapy [216]. Familial MNG and ovarian Sertoli-Leydig cell tumors [216–222] as well as co-occurrence of Sertoli-Leydig cell tumor with TC are highly suggestive of *DICER1* syndrome [223]. The diagnosis of poorly differentiated thyroid carcinoma (PDTC) in childhood or adolescence is a rare event that should also suggest the possibility of a *DICER1* syndrome [224].

#### Pathological Features

**Fig. 4:**
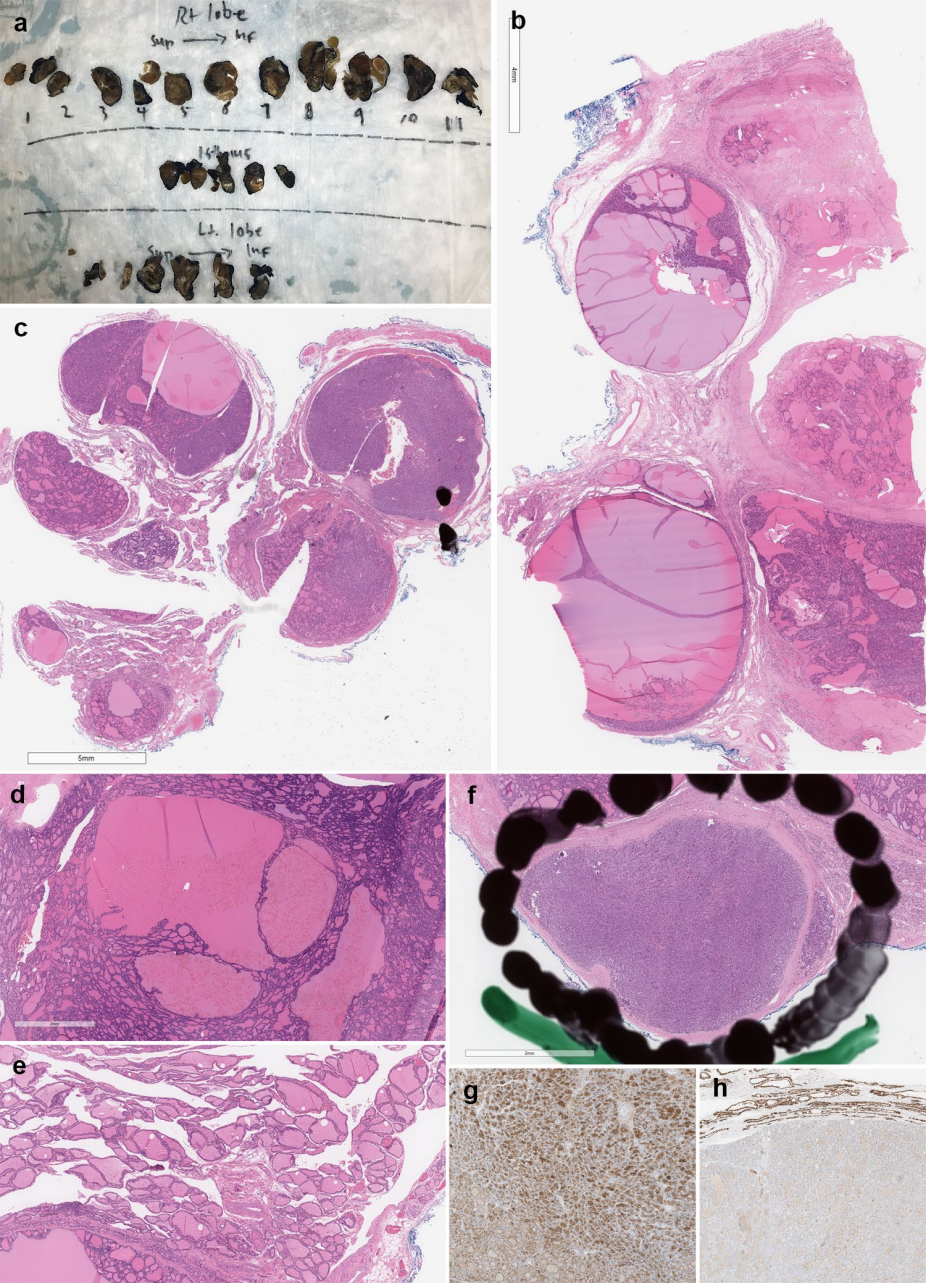
DICER1-related thyroid disease. DICER1-related thyroidectomy specimens are grossly indistinguishable from sporadic manifestations of multinodular goiter (**a**; Lt refers to left; Rt refers to right; Sup refers to superior; Inf refers to inferior). Careful assessment of thyroid nodules and the non-lesional thyroid parenchyma provides additional clues to the possibility of DICER1-related thyroid disease. This composite photomicrograph illustrates features of thyroid pathology identified in a young adult patient with pathogenic germline *DICER1* mutation. The thyroid gland shows multifocal follicular adenomas with intrafollicular centripetal papillary projections, which are also known as papillary adenomas (**b**–**c**). Although papillary adenomas tend to manifest with clinical or subclinical hyperthyroidism; DICER1-related papillary adenomas are seen in association with euthyroid states. In addition, follicular-patterned thyroid neoplasms including follicular adenomas and follicular variant papillary thyroid carcinomas are identified (**c**, **f**). The nontumorous thyroid gland shows variable involutional changes characterized by dilated macro-follicles (**e**). Papillary thyroid carcinomas account for the vast majority of malignant thyroid nodules in DICER1-related thyroid disease. Encapsulated follicular variant papillary microcarcinoma with tumor capsular invasion is illustrated (**f**) along with HBME1 immunoreactivity (**g**) and reduced membranous CD56 expression (**h**)

MNG associated with *DICER1* syndrome includes multiple and bilateral conventional follicular nodular proliferations, well-circumscribed adenomas, and/or nodules displaying intrafollicular centripedal papillary growth (so-called papillary hyperplasia or papillary adenoma) without nuclear features of papillary thyroid carcinoma [225] (Fig. 4). Most TCs are well-differentiated forms, mainly the follicular variant of PTC and minimally invasive FTC. [117]. In some cases, PTC has been described appearing within a follicular nodule [216] suggesting a stepwise progression of malignancy. Follicular cells of benign and malignant nodules (including papillary hyperplasias) sometimes show some nuclear features of PTC (intermediate-type nuclei), fitting with a carcinogenic process different from the classical pathway towards PTC or FTC [9]; for this reason, the criteria for the diagnosis of PTC must be particularly strict in this setting. Rare cases of solid/trabecular variant of PTC [226] and PDTC (defined by the Turin consensus), have also been described in this syndrome [224]. One of the characteristics of DICER1-related thyroid disease is the high frequency of involutional change in the non-tumorous thyroid parenchyma in the absence of clinical or subclinial hyperthyroidism [225].

**Fig. 5:**
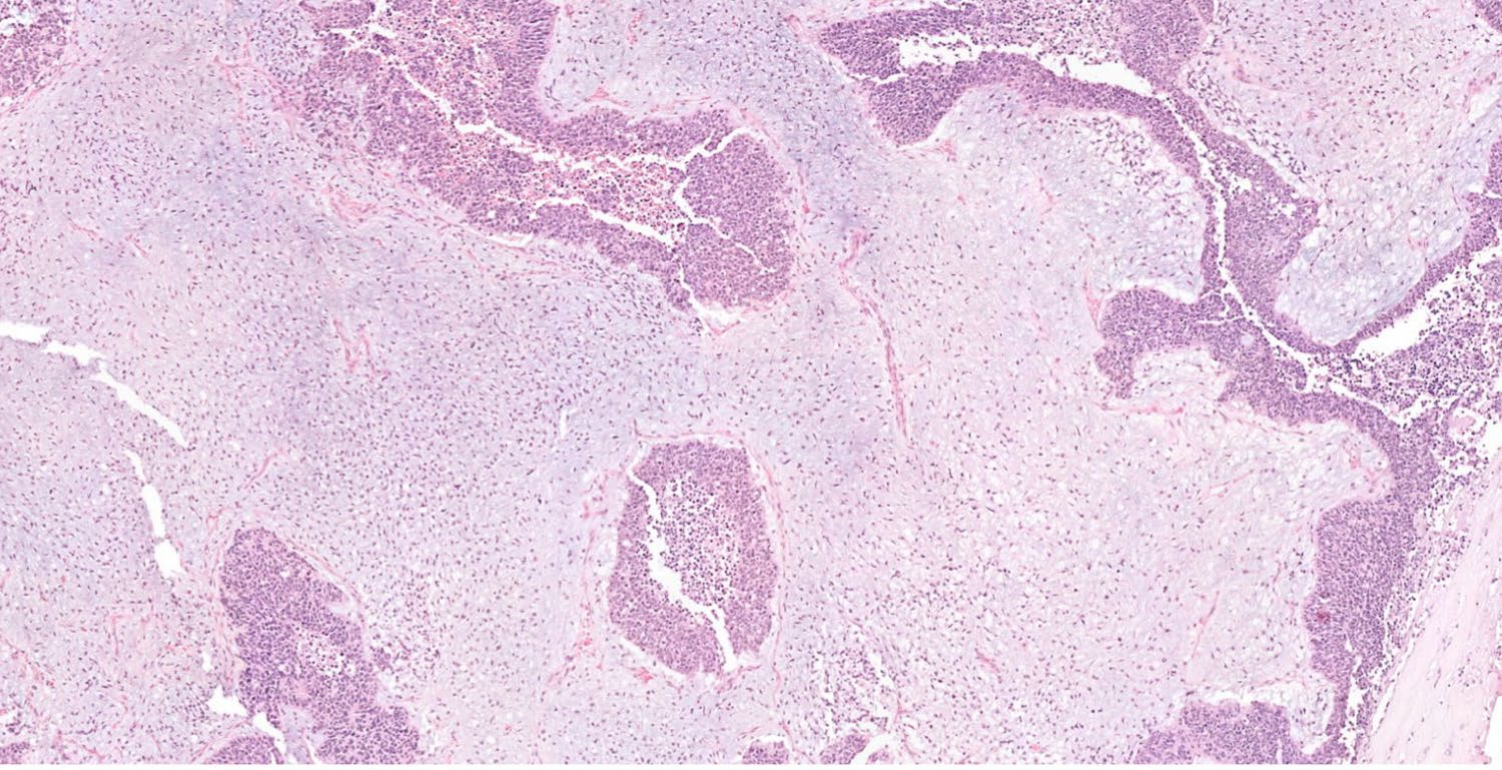
Thyroblastoma. In this area, the tumor is composed of a small cell undifferentiated/immature epithelial component, and a stromal chondroid component (image courtesy of Dr. Catarina Eloy, Porto, Portugal)

A rare malignant teratoid tumor of the thyroid recently designated as “thyroblastoma” [227] has been described as having somatic pathogenic *DICER1* variants but not associated with pathogenic germline *DICER1* variants [227–230] (Fig. 5). This neoplasm is characterized by a triphasic pattern combining TTF1+/PAX8+ primitive teratoid follicle-like glands admixed with neuroepithelial-like and fetal tubule-like components, with a second primitive small cell component, as well as a third cellular stroma with frequent rabdomyoblastic differentiation [227].

#### Genetic Features

*DICER1* (14q32.13) encodes a protein possessing an RNA helicase motif containing a DEXH box in its amino terminus and an RNA motif in the carboxy terminus. The encoded protein functions as a ribonuclease and is required by the RNA interference and small temporal RNA (stRNA) pathways to produce the active small RNA component that represses gene expression. Dicer is a type III cytoplasmic endoribonuclease that is involved in the maturation of several classes of small non-coding RNAs, such as microRNAs [210, 231]. Most individuals with *DICER1* syndrome have a germline loss-of-function *DICER1* mutation with a second tumor-specific missense mutation in the RNase IIIb domain; these tumor-specific RNase IIIb missense mutations usually involve one of five hot spot codons (E1705, S1709, G1809, D1810, and E1813) [208, 210, 232]. Individuals with 14q32 deletions that encompass the *DICER1* locus are also associated with an increased risk for *DICER1*-related tumor development [233].

*DICER1* mutations are rare in sporadic PTC [234]. Somatic mutations in the *DICER1* gene have been detected in two of four cases of macrofollicular variant of FTC [235]. Additional somatic RNase IIIb mutations have been detected in each of two benign follicular nodules from two patients carrying a germline *DICER1* (hot spot) mutation [225]. Another study has also found somatic *DICER1* hot spot mutations in both benign and malignant nodules from *DICER1* carriers, including different somatic *DICER1* mutations from different nodules from the same thyroid gland [117]. In a series of 40 adolescent-onset PTC cases, two somatic *DICER1* alterations were exclusively detected in each of the two PTC cases that lacked the molecular alterations typical of this tumor type (*BRAF*, *HRAS*, *KRAS*, *NRAS*, *RET*, and *PAX8*) [225]. In a similar way, no characteristic mutations of FTC or PTC (including *TERT*) have been reported in a well-differentiated thyroid carcinoma, not otherwise specified (NOS) presenting in a young girl with MNG, botryoid rhabdomyosarcoma, and pathogenic *DICER1* mutation [236]. Childhood- and adolescent-onset PDTCs are genetically distinct from adult-onset PDTCs; they are associated with somatic *DICER1* mutations and less frequently with a germline pathogenic *DICER1* variant, but not with the classic molecular alterations of TC [224]. All these findings support the assumption that *DICER1* syndrome-related TC may develop in a background of MNG, via a stepwise process, involving *DICER1* somatic mutations and additional molecular events, distinct from the classic pathways of TC [9, 117].

### Carney Complex (CNC)

Carney complex (CNC) is an autosomal dominant disease characterized by the following major criteria for diagnosis: peculiar distribution of pigmentation in skin and mucosa (lips, conjunctiva and inner or outer canthi, penile, and vaginal mucosa), multiple myxomas (cutaneous, mucous, cardiac, and/or in the breast), primary pigmented nodular adrenocortical disease (Cushing syndrome), large-cell calcifying Sertoli cell tumors, acromegaly from a growth hormone (GH)-producing pituitary tumor, blue nevus, epithelioid blue nevus, breast ductal adenoma, osteochondromyxoma, and multiple thyroid nodules [237–245]. CNC has also been designated by the acronyms *NAME* (nevi, atrial myxomas, ephelides) [246] and *LAMB* (lentigines, atrial myxoma, blue nevi) [247]. It is impportant to recognize that the Carney triad (pulmonary chondroma, extra-adrenal paraganglioma, and gastrointestinal stromal tumor) [248, 249] is a different entity. The percentage of thyroid nodules reaches 60% among patients with CNC and 75% among children and adolescents [243, 250].

#### Pathological Features

The thyroid gland shows multiple and bilateral proliferative follicular lesions, including Hürthle cell nodules. There is multinodular hyperplasia, sometimes with peculiar microscopic “follicular adenomatosis” along with multiple follicular adenomas in up to 75% of CNC patients [241, 245, 251]. Follicular adenomatosis [244] is characterized by the presence of multiple encapsulated and uncapsulated follicular thyroid nodules distributed throughout the gland in a manner equivalent to the so-called microadenomas that appear in PHTS (see above). Lymphocytic thyroiditis and hyperthyroidism due to diffuse hyperplasia (Graves’ disease) [252] and toxic adenoma(s) have also been reported [244]. CNC-related toxic adenomas are not different from sporadic toxic adenomas that are characterized by intrafollicular centripedal papillary growth. The term “papillary adenomas” is also applied to these functional benign neoplasms [253, 254]. The papillae in toxic adenomas contain subfollicles and are lined by basally oriented nuclei with no features of papillary thyroid carcinomas. Although multifocal nature of these proliferations should alert the diagnostician to the possibility of a germline susceptibility, one should remember that similar findings can also occur in patients with McCune Albright syndrome, which is caused by postzygotic *GNAS* non-familial genetic mosaicism [255]. The incidence of TC is about 15% [245]. The patients usually develop well-differentiated carcinomas, both FTC and PTC, sometimes after a long history of multiple adenomas [241, 244, 256, 257]. Hürthle cell adenoma has been reported in a boy with CNC [258].

#### Genetic Features

In more than half of cases, CNC is caused by a heterozygous germline pathogenic variant in *PRKAR1A* gene (17q24.2) [245]. *PRKAR1A* gene encodes the regulatory subunit type I alpha of the protein kinase A (PKA, cAMP-dependent protein kinase) enzyme [250]. In a series of 353 patients with CNC, pathogenic germline *PRKAR1A* variants were detected in 73% of patients, with a penetrance close to 100%, and most mutations (82%) led to lack of detectable mutant protein because of non-sense mRNA [243]. The percentage of mutations reaches 80% in those patients with primary pigmented nodular adrenocortical disease (the so-called PPNAD) [243]. In some patients with clinical criteria of CNC without *PRKAR1A* mutation, a second locus has been identified at 2p16 [259], but for the majority of *PRKAR1A*-negative CNC cases the genetic cause is unknown [245].

### Werner Syndrome (WS)

Werner Syndrome (WS) is an autosomal recessive disease with genetic instability and cancer predisposition caused by biallelic *WRN* pathogenic variants [260, 261]. WS (also called *progeria of the adult*) is associated with an acceleration of biological aging and elevated risk of cancer [260]. WS is characterized by premature graying and/or thinning of scalp hair, bilateral ocular cataracts, deep, chronic ulcers around the ankles, and short stature, with symptoms typically starting in the 20s. Additional signs and symptoms include thin limbs, osteoporosis, pinched facial features, voice change, hypogonadism, type 2 diabetes mellitus, soft tissue calcification, atherosclerosis, and cancer [260, 262, 263]. In a systemativc review, the ratio male/female has been higher among Japan-resident WS patients than among patients residing outside Japan (79:58 vs. 23:26, respectively). [264]. The most common malignant neoplasms in patients with WS are thyroid tumors, melanoma, meningioma, soft-tissue sarcomas, leukemia and preleukemic disorders, and osteosarcomas [264]. TC usually appears at a younger age (mean: 34 years), and in Japanese people with WS, the overall incidence of TC is 18% [265, 266].

#### Pathological Features

Among the Japanese population with WS and TC, FTC (48%) was most common, followed by PTC (35%) and anaplastic thyroid carcinoma (ATC) (13%) [266]; in this setting, ATC appears in individuals at a younger age than it does in sporadic ATC, probably due to premature aging.

#### Genetic Features

*WRN* gene (8p12) encodes a multifunctional nuclear protein that is a member of the RecQ family of DNA helicases [267]. WRN protein seems to be involved in DNA repair, recombination, replication, and transcription [261]. Additionaly, WRN protein is implicated in the maintenance of telomeres [268]. In individuals of Japanese descent, PTC has been associated with the c.1105C>T, p.R369* mutation, whereas FTC was associated with the c.3139-1G>C mutation (exon 26 skip), but the mutational spectrum is different between Japanese and Caucasian patients [266]

## Conclusions

Cancer derived from thyroid follicular epithelial cells is common; it represents the most common endocrine malignancy. The molecular features of the sporadic tumors have been clarified in the past decade. However the incidence of familial disease has not been emphasized and is often overlooked in routine practice. In this review, we have summarized a large body of information about both syndromic and non-syndromic familial thyroid carcinomas. In syndromic cases, the morphology of the tumor(s), molecular immunohistochemistry (e.g., PTEN, beta-catenin, SDHB), the findings in the non-tumorous thyroid parenchyma, and other associated lesions may provide insight into the underlying disorder. However, the increasing evidence of familial predisposition to non-syndromic thyroid cancers is raising questions about the importance of genetics and epigenetics. What appears to be “sporadic” in becoming less often truly so and more often an opportunity to identify and understand novel genetic variants that underlie tumorigenesis. Pathologists must be aware of the unusual morphologic features that are harbingers of specific germline susceptibility syndromes and can assist in providing information to uncover biomarkers that will facilitate screening and early detection to prevent aggressive disease.

## References

[1] Maxwell EL, Hall FT, Freeman JL (2004). Familial non-medullary thyroid cancer: a matched-case control study. Laryngoscope..

[2] Charkes ND (2006). On the prevalence of familial nonmedullary thyroid cancer in multiply affected kindreds. Thyroid..

[3] Sippel RS, Caron NR, Clark OH (2007). An evidence-based approach to familial nonmedullary thyroid cancer: screening, clinical management, and follow-up. World J Surg..

[4] Ito Y, Kakudo K, Hirokawa M, Fukushima M, Yabuta T, Tomoda C, Inoue H, Kihara M, Higashiyama T, Uruno T, Takamura Y, Miya A, Kobayashi K, Matsuzuka F, Miyauchi A (2009). Biological behavior and prognosis of familial papillary thyroid carcinoma. Surgery..

[5] Moses W, Weng J, Kebebew E (2011). Prevalence, clinicopathologic features, and somatic genetic mutation profile in familial versus sporadic nonmedullary thyroid cancer. Thyroid..

[6] Robenshtok E, Tzvetov G, Grozinsky-Glasberg S, Shraga-Slutzky I, Weinstein R, Lazar L, Serov S, Singer J, Hirsch D, Shimon I, Benbassat C (2011). Clinical characteristics and outcome of familial nonmedullary thyroid cancer: a retrospective controlled study. Thyroid..

[7] Park YJ, Ahn HY, Choi HS, Kim KW, Park DJ, Cho BY (2012). The long-term outcomes of the second generation of familial nonmedullary thyroid carcinoma are more aggressive than sporadic cases. Thyroid..

[8] Mazeh H, Sippel RS (2013). Familial nonmedullary thyroid carcinoma. Thyroid..

[9] Cameselle-Teijeiro JM, Eloy C, Amendoeira I, Soares P, Caneiro-Gómez J, Melo M, Sobrinho-Simoes M, Cameselle-Teijeiro JM, Eloy C, Sobrinho-Simoes M (2018). Rare Familial Tumours. Rare tumors of the thyroid gland: diagnosis and WHO classification.

[10] Kim YS, Seo M, Park SH, Ju SY, Kim ES (2020). Should Total Thyroidectomy Be Recommended for Patients with Familial Non-medullary Thyroid Cancer?. World J Surg..

[11] Mete O, Asa SL, Volante M, Papotti M, Mete O, Asa SL (2016). Malignant follicular epithelial proliferations. Endocrine Pathology.

[12] Robinson DW, Orr TG (1955). Carcinoma of the thyroid and other diseases of the thyroid in identical twins. AMA Arch Surg..

[13] Bevan S, Pal T, Greenberg CR, Green H, Wixey J, Bignell G, Narod SA, Foulkes WD, Stratton MR, Houlston RS (2001). A comprehensive analysis of MNG1, TCO1, fPTC, PTEN, TSHR, and TRKA in familial nonmedullary thyroid cancer: confirmation of linkage to TCO1. J Clin Endocrinol Metab..

[14] Mazeh H, Benavidez J, Poehls JL, Youngwirth L, Chen H, Sippel RS (2012). In patients with thyroid cancer of follicular cell origin, a family history of nonmedullary thyroid cancer in one first-degree relative is associated with more aggressive disease. Thyroid..

[15] Capezzone M, Fralassi N, Secchi C, Cantara S, Brilli L, Pilli T, Maino F, Forleo R, Pacini F, Cevenini G, Cartocci A, Castagna MG (2020). Long-Term Clinical Outcome in Familial and Sporadic Papillary Thyroid Carcinoma. Eur Thyroid J..

[16] Musholt TJ, Musholt PB, Petrich T, Oetting G, Knapp WH, Klempnauer J (2000). Familial papillary thyroid carcinoma: genetics, criteria for diagnosis, clinical features, and surgical treatment. World J Surg..

[17] Capezzone M, Robenshtok E, Cantara S, Castagna MG. Familial non-medullary thyroid cancer: a critical review. J Endocrinol Invest. 2020 Oct 6. 10.1007/s40618-020-01435-x. Epub ahead of print. PMID: 33025555. 10.1007/s40618-020-01435-x PMC8049908 33025555

[18] Dotto J, Nosé V (2008). Familial thyroid carcinoma: a diagnostic algorithm. Adv Anat Pathol..

[19] Hemminki K, Eng C, Chen B (2005). Familial risks for nonmedullary thyroid cancer. J Clin Endocrinol Metab..

[20] Nixon IJ, Suárez C, Simo R, Sanabria A, Angelos P, Rinaldo A, Rodrigo JP, Kowalski LP, Hartl DM, Hinni ML, Shah JP, Ferlito A (2016). The impact of family history on non-medullary thyroid cancer. Eur J Surg Oncol..

[21] Muallem Kalmovich L, Musholt TJ, Musholt PB, Petrich T, Oetting G, Knapp WH, Klempnauer J (2000). Familial papillary thyroid carcinoma: genetics, criteria for diagnosis, clinical features, and surgical treatment. World J Surg..

[22] Loh KC, Lo JC, Greenspan FS, Miller TR, Yeo PP. Familial papillary thyroid cancer: a case report. Ann Acad Med Singap. 1997;26(4):503–6. 9395820

[23] Alsanea O, Wada N, Ain K, Wong M, Taylor K, Ituarte PH, Treseler PA, Weier HU, Freimer N, Siperstein AE, Duh QY, Takami H, Clark OH. Is familial non-medullary thyroid carcinoma more aggressive than sporadic thyroid cancer? A multicenter series. Surgery. 2000;128(6):1043–50;discussion 1050–1. 10.1067/msy.2000.110848 11114641

[24] Sturgeon C, Clark OH (2005). Familial nonmedullary thyroid cancer. Thyroid..

[25] Tavarelli M, Russo M, Terranova R, Scollo C, Spadaro A, Sapuppo G, Malandrino P, Masucci R, Squatrito S, Pellegriti G (2015). Familial Non-Medullary Thyroid Cancer Represents an Independent Risk Factor for Increased Cancer Aggressiveness: A Retrospective Analysis of 74 Families. Front Endocrinol (Lausanne)..

[26] Uchino S, Noguchi S, Kawamoto H, Yamashita H, Watanabe S, Yamashita H, Shuto S (2002). Familial nonmedullary thyroid carcinoma characterized by multifocality and a high recurrence rate in a large study population. World J Surg..

[27] McDonald TJ, Driedger AA, Garcia BM, Van Uum SH, Rachinsky I, Chevendra V, Breadner D, Feinn R, Walsh SJ, Malchoff CD (2011). Familial papillary thyroid carcinoma: a retrospective analysis. J Oncol..

[28] Capezzone M, Marchisotta S, Cantara S, Busonero G, Brilli L, Pazaitou-Panayiotou K, Carli AF, Caruso G, Toti P, Capitani S, Pammolli A, Pacini F (2008). Familial non-medullary thyroid carcinoma displays the features of clinical anticipation suggestive of a distinct biological entity. Endocr Relat Cancer..

[29] Klubo-Gwiezdzinska J, Yang L, Merkel R, Patel D, Nilubol N, Merino MJ, Skarulis M, Sadowski SM, Kebebew E (2017). Results of Screening in Familial Non-Medullary Thyroid Cancer. Thyroid..

[30] Leprat F, Bonichon F, Guyot M, Trouette H, Trojani M, Vergnot V, Longy M, Belleannée G, de Mascarel A, Roger P (1999). Familial non-medullary thyroid carcinoma: pathology review in 27 affected cases from 13 French families. Clin Endocrinol (Oxf)..

[31] Lupoli G, Vitale G, Caraglia M, Fittipaldi MR, Abbruzzese A, Tagliaferri P, Bianco AR (1999). Familial papillary thyroid microcarcinoma: a new clinical entity. Lancet..

[32] Lesueur F, Stark M, Tocco T, Ayadi H, Delisle MJ, Goldgar DE, Schlumberger M, Romeo G, Canzian F (1999). Genetic heterogeneity in familial nonmedullary thyroid carcinoma: exclusion of linkage to RET, MNG1, and TCO in 56 families. NMTC Consortium. J Clin Endocrinol Metab..

[33] Suh I, Filetti S, Vriens MR, Guerrero MA, Tumino S, Wong M, Shen WT, Kebebew E, Duh QY, Clark OH (2009). Distinct loci on chromosome 1q21 and 6q22 predispose to familial nonmedullary thyroid cancer: a SNP array-based linkage analysis of 38 families. Surgery..

[34] Muallem Kalmovich L, Jabarin B, Koren S, Or K, Marcus E, Tkacheva I, Benbassat C, Steinschneider M. Is Familial Nonmedullary Thyroid Cancer A More Aggressive Type of Thyroid Cancer? Laryngoscope. 2020 Aug 6. 10.1002/lary.28989. Epub ahead of print. PMID: 32761812. 10.1002/lary.28989 32761812

[35] Grossman RF, Tu SH, Duh QY, Siperstein AE, Novosolov F, Clark OH. Familial nonmedullary thyroid cancer. An emerging entity that warrants aggressive treatment. Arch Surg. 1995;130(8):892–7; discussion 898–9. 10.1001/archsurg.1995.01430080094015 7632152

[36] Burgess JR, Duffield A, Wilkinson SJ, Ware R, Greenaway TM, Percival J, Hoffman L (1997). Two families with an autosomal dominant inheritance pattern for papillary carcinoma of the thyroid. J Clin Endocrinol Metab..

[37] Pal T, Vogl FD, Chappuis PO, Tsang R, Brierley J, Renard H, Sanders K, Kantemiroff T, Bagha S, Goldgar DE, Narod SA, Foulkes WD (2001). Increased risk for nonmedullary thyroid cancer in the first degree relatives of prevalent cases of nonmedullary thyroid cancer: a hospital-based study. J Clin Endocrinol Metab..

[38] Wang X, Cheng W, Li J, Su A, Wei T, Liu F, Zhu J (2015). Endocrine tumours: familial nonmedullary thyroid carcinoma is a more aggressive disease: a systematic review and meta-analysis. Eur J Endocrinol..

[39] Zhang Q, Yang S, Meng XY, Chen G, Pang RZ (2016). Clinical Analysis of Familial Nonmedullary Thyroid Carcinoma. World J Surg..

[40] Canzian F, Amati P, Harach HR, Kraimps JL, Lesueur F, Barbier J, Levillain P, Romeo G, Bonneau D. A gene predisposing to familial thyroid tumors with cell oxyphilia maps to chromosome 19p13.2. Am J Hum Genet. 1998;63(6):1743–8. 10.1086/302164 PMC1377646 9837827

[41] Harach HR, Lesueur F, Amati P, Brown A, Canzian F, Kraimps JL, Levillain P, Menet E, Romeo G, Bonneau D. Histology of familial thyroid tumours linked to a gene mapping to chromosome 19p13.2. J Pathol. 1999;189(3):387–93. 10.1002/(SICI)1096-9896(199911)189:3<387::AID-PATH443>3.0.CO;2-S 10547601

[42] McKay JD, Thompson D, Lesueur F, Stankov K, Pastore A, Watfah C, Strolz S, Riccabona G, Moncayo R, Romeo G, Goldgar DE (2004). Evidence for interaction between the TCO and NMTC1 loci in familial non-medullary thyroid cancer. J Med Genet..

[43] Máximo V, Botelho T, Capela J, Soares P, Lima J, Taveira A, Amaro T, Barbosa AP, Preto A, Harach HR, Williams D, Sobrinho-Simões M (2005). Somatic and germline mutation in GRIM-19, a dual function gene involved in mitochondrial metabolism and cell death, is linked to mitochondrion-rich (Hurthle cell) tumours of the thyroid. Br J Cancer..

[44] Lee YM, Yoon JH, Yi O, Sung TY, Chung KW, Kim WB, Hong SJ (2014). Familial history of non-medullary thyroid cancer is an independent prognostic factor for tumor recurrence in younger patients with conventional papillary thyroid carcinoma. J Surg Oncol..

[45] Triponez F, Wong M, Sturgeon C, Caron N, Ginzinger DG, Segal MR, Kebebew E, Duh QY, Clark OH (2006). Does familial non-medullary thyroid cancer adversely affect survival?. World J Surg..

[46] Sung TY, Lee YM, Yoon JH, Chung KW, Hong SJ (2015). Surgical Management of Familial Papillary Thyroid Microcarcinoma: A Single Institution Study of 94 Cases. World J Surg..

[47] Cavaco BM, Batista PF, Sobrinho LG, Leite V. Mapping a new familial thyroid epithelial neoplasia susceptibility locus to chromosome 8p23.1-p22 by high-density single-nucleotide polymorphism genome-wide linkage analysis. J Clin Endocrinol Metab. 2008;93(11):4426–30. 10.1210/jc.2008-0449 18765515

[48] Prazeres H, Torres J, Soares P, Sobrinho-Simões M (2010). The familial counterparts of follicular cell–derived thyroid tumors. Int J Surg Pathol..

[49] Peiling Yang S, Ngeow J (2016). Familial non-medullary thyroid cancer: unraveling the genetic maze. Endocr Relat Cancer..

[50] Hińcza K, Kowalik A, Kowalska A (2019). Current Knowledge of Germline Genetic Risk Factors for the Development of Non-Medullary Thyroid Cancer. Genes (Basel)..

[51] Srivastava A, Kumar A, Giangiobbe S, Bonora E, Hemminki K, Försti A, Bandapalli OR (2019). Whole Genome Sequencing of Familial Non-Medullary Thyroid Cancer Identifies Germline Alterations in MAPK/ERK and PI3K/AKT Signaling Pathways. Biomolecules..

[52] Bonora E, Rizzato C, Diquigiovanni C, Oudot-Mellakh T, Campa D, Vargiolu M, Guedj M; NMTC Consortium, McKay JD, Romeo G, Canzian F, Lesueur F. The FOXE1 locus is a major genetic determinant for familial nonmedullary thyroid carcinoma. Int J Cancer. 2014;134(9):2098–107. 10.1002/ijc.28543 24127282

[53] Mancikova V, Cruz R, Inglada-Pérez L, Fernández-Rozadilla C, Landa I, Cameselle-Teijeiro J, Celeiro C, Pastor S, Velázquez A, Marcos R, Andía V, Álvarez-Escolá C, Meoro A, Schiavi F, Opocher G, Quintela I, Ansede-Bermejo J, Ruiz-Ponte C, Santisteban P, Robledo M, Carracedo A. Thyroid cancer GWAS identifies 10q26.12 and 6q14.1 as novel susceptibility loci and reveals genetic heterogeneity among populations. Int J Cancer. 2015;137(8):1870–8. 10.1002/ijc.29557 25855579

[54] Pereira JS, da Silva JG, Tomaz RA, Pinto AE, Bugalho MJ, Leite V, Cavaco BM (2015). Identification of a novel germline FOXE1 variant in patients with familial non-medullary thyroid carcinoma (FNMTC). Endocrine..

[55] Ngan ES, Lang BH, Liu T, Shum CK, So MT, Lau DK, Leon TY, Cherny SS, Tsai SY, Lo CY, Khoo US, Tam PK, Garcia-Barceló MM. A germline mutation (A339V) in thyroid transcription factor-1 (TITF-1/NKX2.1) in patients with multinodular goiter and papillary thyroid carcinoma. J Natl Cancer Inst. 2009;101(3):162–75. 10.1093/jnci/djn471 19176457

[56] Liao S, Song W, Liu Y, Deng S, Liang Y, Tang Z, Huang J, Dong D, Xu G (2013). Familial multinodular goiter syndrome with papillary thyroid carcinomas: mutational analysis of the associated genes in 5 cases from 1 Chinese family. BMC Endocr Disord..

[57] He H, Bronisz A, Liyanarachchi S, Nagy R, Li W, Huang Y, Akagi K, Saji M, Kula D, Wojcicka A, Sebastian N, Wen B, Puch Z, Kalemba M, Stachlewska E, Czetwertynska M, Dlugosinska J, Dymecka K, Ploski R, Krawczyk M, Morrison PJ, Ringel MD, Kloos RT, Jazdzewski K, Symer DE, Vieland VJ, Ostrowski M, Jarząb B, de la Chapelle A (2013). SRGAP1 is a candidate gene for papillary thyroid carcinoma susceptibility. J Clin Endocrinol Metab..

[58] Ye F, Gao H, Xiao L, Zuo Z, Liu Y, Zhao Q, Chen H, Feng W, Fu B, Sun L, Jiang X, He D, Jiang H, Yang M, Li L, Chen F, Liu X, Li S, Li Z, Jiang Y, Cheng L, Bu H (2019). Whole exome and target sequencing identifies MAP2K5 as novel susceptibility gene for familial non-medullary thyroid carcinoma. Int J Cancer..

[59] Bakhsh A, Kirov G, Gregory JW, Williams ED, Ludgate M (2006). A new form of familial multi-nodular goitre with progression to differentiated thyroid cancer. Endocr Relat Cancer..

[60] Bakhsh AD, Ladas I, Hamshere ML, Bullock M, Kirov G, Zhang L, Taylor PN, Gregory JW, Scott-Coombes D, Völzke H, Teumer A, Mantripragada K, Williams ED, Clifton-Bligh RJ, Williams NM, Ludgate ME (2018). An InDel in Phospholipase-C-B-1 Is Linked with Euthyroid Multinodular Goiter. Thyroid..

[61] Williams D (2020). Inherited thyroid tumours. Endocrine..

[62] Gara SK, Jia L, Merino MJ, Agarwal SK, Zhang L, Cam M, Patel D, Kebebew E (2015). Germline HABP2 Mutation Causing Familial Nonmedullary Thyroid Cancer. N Engl J Med..

[63] Zhang T, Xing M. HABP2 G534E Mutation in Familial Nonmedullary Thyroid Cancer. J Natl Cancer Inst. 2016;108(6):djv415. 10.1093/jnci/djv415 PMC4909127 26832773

[64] Pasquali D, Torella A, Accardo G, Esposito D, Del Vecchio Blanco F, Salvatore D, Sabatino P, Pacini F, Barbato F, Castagna MG, Cantara S, Nigro V. BROX haploinsufficiency in familial nonmedullary thyroid cancer. J Endocrinol Invest. 2020 May 8. 10.1007/s40618-020-01286-6. Epub ahead of print. PMID: 32385852. 10.1007/s40618-020-01286-6 32385852

[65] Wilson TL, Hattangady N, Lerario AM, Williams C, Koeppe E, Quinonez S, Osborne J, Cha KB, Else T (2017). A new POT1 germline mutation-expanding the spectrum of POT1-associated cancers. Fam Cancer..

[66] Potrony M, Puig-Butille JA, Ribera-Sola M, Iyer V, Robles-Espinoza CD, Aguilera P, Carrera C, Malvehy J, Badenas C, Landi MT, Adams DJ, Puig S (2019). POT1 germline mutations but not TERT promoter mutations are implicated in melanoma susceptibility in a large cohort of Spanish melanoma families. Br J Dermatol..

[67] Richard MA, Lupo PJ, Morton LM, Yasui YA, Sapkota YA, Arnold MA, Aubert G, Neglia JP, Turcotte LM, Leisenring WM, Sampson JN, Chanock SJ, Hudson MM, Armstrong GT, Robison LL, Bhatia S, Gramatges MM (2020). Genetic variation in POT1 and risk of thyroid subsequent malignant neoplasm: A report from the Childhood Cancer Survivor Study. PLoS One..

[68] Srivastava A, Miao B, Skopelitou D, Kumar V, Kumar A, Paramasivam N, Bonora E, Hemminki K, Försti A, Bandapalli OR (2020). A Germline Mutation in the POT1 Gene Is a Candidate for Familial Non-Medullary Thyroid Cancer. Cancers (Basel)..

[69] Orois A, Gara SK, Mora M, Halperin I, Martínez S, Alfayate R, Kebebew E, Oriola J (2019). NOP53 as A Candidate Modifier Locus for Familial Non-Medullary Thyroid Cancer. Genes (Basel)..

[70] Zhao Y, Yu T, Chen L, Xie D, Wang F, Fu L, Cheng C, Li Y, Zhu X, Miao G (2020). A Germline CHEK2 Mutation in a Family with Papillary Thyroid Cancer. Thyroid..

[71] Gąsior-Perczak D, Kowalik A, Walczyk A, Siołek M, Gruszczyński K, Pałyga I, Mikina E, Trybek T, Kopczyński J, Mężyk R, Góźdź S, Kowalska A (2019). Coexisting Germline CHEK2 and Somatic BRAFV600E Mutations in Papillary Thyroid Cancer and Their Association with Clinicopathological Features and Disease Course. Cancers (Basel)..

[72] Siołek M, Cybulski C, Gąsior-Perczak D, Kowalik A, Kozak-Klonowska B, Kowalska A, Chłopek M, Kluźniak W, Wokołorczyk D, Pałyga I, Walczyk A, Lizis-Kolus K, Sun P, Lubiński J, Narod SA, Góźdż S (2015). CHEK2 mutations and the risk of papillary thyroid cancer. Int J Cancer..

[73] Bonora E, Evangelisti C, Bonichon F, Tallini G, Romeo G (2006). Novel germline variants identified in the inner mitochondrial membrane transporter TIMM44 and their role in predisposition to oncocytic thyroid carcinomas. Br J Cancer..

[74] Sarquis M, Moraes DC, Bastos-Rodrigues L, Azevedo PG, Ramos AV, Reis FV, Dande PV, Paim I, Friedman E, De Marco L (2020). Germline Mutations in Familial Papillary Thyroid Cancer. Endocr Pathol..

[75] Prazeres HJ, Rodrigues F, Soares P, Naidenov P, Figueiredo P, Campos B, Lacerda M, Martins TC. Loss of heterozygosity at 19p13.2 and 2q21 in tumours from familial clusters of non-medullary thyroid carcinoma. Fam Cancer. 2008;7(2):141–9. 10.1007/s10689-007-9160-x 17823852

[76] Stankov K, Pastore A, Toschi L, McKay J, Lesueur F, Kraimps JL, Bonneau D, Gibelin H, Levillain P, Volante M, Papotti M, Romeo G. Allelic loss on chromosomes 2q21 and 19p 13.2 in oxyphilic thyroid tumors. Int J Cancer. 2004;111(3):463–7. 10.1002/ijc.20259 15221978

[77] He H, Nagy R, Liyanarachchi S, Jiao H, Li W, Suster S, Kere J, de la Chapelle A (2009). A susceptibility locus for papillary thyroid carcinoma on chromosome 8q24. Cancer Res..

[78] Malchoff CD, Sarfarazi M, Tendler B, Forouhar F, Whalen G, Joshi V, Arnold A, Malchoff DM (2000). Papillary thyroid carcinoma associated with papillary renal neoplasia: genetic linkage analysis of a distinct heritable tumor syndrome. J Clin Endocrinol Metab..

[79] Bignell GR, Canzian F, Shayeghi M, Stark M, Shugart YY, Biggs P, Mangion J, Hamoudi R, Rosenblatt J, Buu P, Sun S, Stoffer SS, Goldgar DE, Romeo G, Houlston RS, Narod SA, Stratton MR, Foulkes WD (1997). Familial nontoxic multinodular thyroid goiter locus maps to chromosome 14q but does not account for familial nonmedullary thyroid cancer. Am J Hum Genet..

[80] McKay JD, Lesueur F, Jonard L, Pastore A, Williamson J, Hoffman L, Burgess J, Duffield A, Papotti M, Stark M, Sobol H, Maes B, Murat A, Kääriäinen H, Bertholon-Grégoire M, Zini M, Rossing MA, Toubert ME, Bonichon F, Cavarec M, Bernard AM, Boneu A, Leprat F, Haas O, Lasset C, Schlumberger M, Canzian F, Goldgar DE, Romeo G (2001). Localization of a susceptibility gene for familial nonmedullary thyroid carcinoma to chromosome 2q21. Am J Hum Genet..

[81] Cavaco BM, Batista PF, Martins C, Banito A, do Rosário F, Limbert E, Sobrinho LG, Leite V. Familial non-medullary thyroid carcinoma (FNMTC): analysis of fPTC/PRN, NMTC1, MNG1 and TCO susceptibility loci and identification of somatic BRAF and RAS mutations. Endocr Relat Cancer. 2008 Mar;15(1):207–15. 10.1677/ERC-07-0214 18310288

[82] Matsuse M, Takahashi M, Mitsutake N, Nishihara E, Hirokawa M, Kawaguchi T, Rogounovitch T, Saenko V, Bychkov A, Suzuki K, Matsuo K, Tajima K, Miyauchi A, Yamada R, Matsuda F, Yamashita S (2011). The FOXE1 and NKX2-1 loci are associated with susceptibility to papillary thyroid carcinoma in the Japanese population. J Med Genet..

[83] Gudmundsson J, Sulem P, Gudbjartsson DF, Jonasson JG, Sigurdsson A, Bergthorsson JT, He H, Blondal T, Geller F, Jakobsdottir M, Magnusdottir DN, Matthiasdottir S, Stacey SN, Skarphedinsson OB, Helgadottir H, Li W, Nagy R, Aguillo E, Faure E, Prats E, Saez B, Martinez M, Eyjolfsson GI, Bjornsdottir US, Holm H, Kristjansson K, Frigge ML, Kristvinsson H, Gulcher JR, Jonsson T, Rafnar T, Hjartarsson H, Mayordomo JI, de la Chapelle A, Hrafnkelsson J, Thorsteinsdottir U, Kong A, Stefansson K. Common variants on 9q22.33 and 14q13.3 predispose to thyroid cancer in European populations. Nat Genet. 2009;41(4):460–4. 10.1038/ng.339 PMC3664837 19198613

[84] Landa I, Ruiz-Llorente S, Montero-Conde C, Inglada-Pérez L, Schiavi F, Leskelä S, Pita G, Milne R, Maravall J, Ramos I, Andía V, Rodríguez-Poyo P, Jara-Albarrán A, Meoro A, del Peso C, Arribas L, Iglesias P, Caballero J, Serrano J, Picó A, Pomares F, Giménez G, López-Mondéjar P, Castello R, Merante-Boschin I, Pelizzo MR, Mauricio D, Opocher G, Rodríguez-Antona C, González-Neira A, Matías-Guiu X, Santisteban P, Robledo M (2009). The variant rs1867277 in FOXE1 gene confers thyroid cancer susceptibility through the recruitment of USF1/USF2 transcription factors. PLoS Genet..

[85] Bullock M, Duncan EL, O'Neill C, Tacon L, Sywak M, Sidhu S, Delbridge L, Learoyd D, Robinson BG, Ludgate M, Clifton-Bligh RJ (2012). Association of FOXE1 polyalanine repeat region with papillary thyroid cancer. J Clin Endocrinol Metab..

[86] Bychkov A, Saenko V, Nakashima M, Mitsutake N, Rogounovitch T, Nikitski A, Orim F, Yamashita S (2013). Patterns of FOXE1 expression in papillary thyroid carcinoma by immunohistochemistry. Thyroid..

[87] Cantara S, Capuano S, Formichi C, Pisu M, Capezzone M, Pacini F. Lack of germline A339V mutation in thyroid transcription factor-1 (TITF-1/NKX2.1) gene in familial papillary thyroid cancer. Thyroid Res. 2010;3(1):4. 10.1186/1756-6614-3-4 PMC2930630 20701785

[88] Simeone P, Alberti S. RE: HABP2 G534E Mutation in Familial Nonmedullary Thyroid Cancer. J Natl Cancer Inst. 2016;108(8):djw143. 10.1093/jnci/djw143 PMC5017948 27418631

[89] Gerhard GS, Bann DV, Broach J, Goldenberg D (2017). Pitfalls of exome sequencing: a case study of the attribution of HABP2 rs7080536 in familial non-medullary thyroid cancer. NPJ Genom Med..

[90] Cazabat L, Terray A, de Mazancourt P, Ropers J, Groussin L, Raffin-Sanson ML (2017). Next Generation Sequencing and Association Studies in Familial Nonmedullary Thyroid Carcinoma: Let's Choose Appropriate Controls. Eur Thyroid J..

[91] Zhao X, Li X, Zhang X (2015). HABP2 Mutation and Nonmedullary Thyroid Cancer. N Engl J Med..

[92] Ruiz-Ferrer M, Fernández RM, Navarro E, Antiñolo G, Borrego S (2016). G534E Variant in HABP2 and Nonmedullary Thyroid Cancer. Thyroid..

[93] Tomsic J, Fultz R, Liyanarachchi S, He H, Senter L, de la Chapelle A (2016). HABP2 G534E Variant in Papillary Thyroid Carcinoma. PLoS One..

[94] Weeks AL, Wilson SG, Ward L, Goldblatt J, Hui J, Walsh JP (2016). HABP2 germline variants are uncommon in familial nonmedullary thyroid cancer. BMC Med Genet..

[95] Cantara S, Marzocchi C, Castagna MG, Pacini F (2017). HABP2 G534E variation in familial non-medullary thyroid cancer: an Italian series. J Endocrinol Invest..

[96] de Mello LEB, Araujo AN, Alves CX, de Paiva FJP, Brandão-Neto J, Cerutti JM (2017). The G534E variant in HABP2 is not associated with increased risk of familial nonmedullary thyroid cancer in Brazilian Kindreds. Clin Endocrinol (Oxf)..

[97] Kern B, Coppin L, Romanet P, Crépin M, Szuster I, Renaud F, Leteurtre E, Frénois F, Wemeau JL, Carnaille B, Cardot-Bauters C, Do Cao C, Pigny P (2017). Multiple HABP2 variants in familial papillary thyroid carcinoma: Contribution of a group of "thyroid-checked" controls. Eur J Med Genet..

[98] Pinheiro M, Drigo SA, Tonhosolo R, Andrade SCS, Marchi FA, Jurisica I, Kowalski LP, Achatz MI, Rogatto SR. HABP2 p.G534E variant in patients with family history of thyroid and breast cancer. Oncotarget. 2017;8(25):40896–905. 10.18632/oncotarget.16639 PMC5522276 28402931

[99] Colombo C, Fugazzola L, Muzza M, Proverbio MC, Cirello V (2018). Letter regarding the article: "Multiple HABP2 variants in familial papillary thyroid carcinoma: Contribution of a group of "thyroid-checked" controls" by Kern. Eur J Med Genet..

[100] de Randamie R, Martos-Moreno GÁ, Lumbreras C, Chueca M, Donnay S, Luque M, Regojo RM, Mendiola M, Hardisson D, Argente J, Moreno JC (2018). Frequent and Rare HABP2 Variants Are Not Associated with Increased Susceptibility to Familial Nonmedullary Thyroid Carcinoma in the Spanish Population. Horm Res Paediatr..

[101] Kowalik A, Gąsior-Perczak D, Gromek M, Siołek M, Walczyk A, Pałyga I, Chłopek M, Kopczyński J, Mężyk R, Kowalska A, Góźdź S. The p.G534E variant of HABP2 is not associated with sporadic papillary thyroid carcinoma in a Polish population. Oncotarget. 2017;8(35):58304–8. 10.18632/oncotarget.16870 PMC5601653 28938557

[102] Orois A, Badenas C, Reverter JL, López V, Potrony M, Mora M, Halperin I, Oriola J (2020). Lack of Mutations in POT1 Gene in Selected Families with Familial Non-Medullary Thyroid Cancer. Horm Cancer..

[103] Wang Y, Liyanarachchi S, Miller KE, Nieminen TT, Comiskey DF, Li W, Brock P, Symer DE, Akagi K, DeLap KE, He H, Koboldt DC, de la Chapelle A (2019). Identification of Rare Variants Predisposing to Thyroid Cancer. Thyroid..

[104] Teshiba R, Tajiri T, Sumitomo K, Masumoto K, Taguchi T, Yamamoto K (2013). Identification of a KEAP1 germline mutation in a family with multinodular goitre. PLoS One..

[105] Zhu M, Wen X, Liu X, Wang Y, Liang C, Tu J (2017). Association between 8q24 rs6983267 polymorphism and cancer susceptibility: a meta-analysis involving 170,737 subjects. Oncotarget..

[106] Tong Y, Tang Y, Li S, Zhao F, Ying J, Qu Y, Niu X, Mu D (2020). Cumulative evidence of relationships between multiple variants in 8q24 region and cancer incidence. Medicine (Baltimore)..

[107] Schultz KAP, Stewart DR, Kamihara J, Bauer AJ, Merideth MA, Stratton P, Huryn LA, Harris AK, Doros L, Field A, Carr AG, Dehner LP, Messinger Y, Hill DA. DICER1 Tumor Predisposition. [updated 2020 Apr 30]. In: Adam MP, Ardinger HH, Pagon RA, Wallace SE, Bean LJH, Stephens K, Amemiya A, editors. GeneReviews® [Internet]. Seattle (WA): University of Washington, Seattle; 1993–2020. PMID: 24761742.

[108] Capon F, Tacconelli A, Giardina E, Sciacchitano S, Bruno R, Tassi V, Trischitta V, Filetti S, Dallapiccola B, Novelli G (2000). Mapping a dominant form of multinodular goiter to chromosome Xp22. Am J Hum Genet..

[109] Giardina E, Capon F, D'Apice MR, Amati F, Arturi F, Filetti S, Bonifazi E, Pucci S, Conte C, Novelli G (2002). Mutational analysis of Peroxiredoxin IV: exclusion of a positional candidate for multinodular goitre. BMC Med Genet..

[110] Takahashi T, Nozaki J, Komatsu M, Wada Y, Utsunomiya M, Inoue K, Takada G, Koizumi A. A new locus for a dominant form of multinodular goiter on 3q26.1-q26.3. Biochem Biophys Res Commun. 2001;284(3):650–4. 10.1006/bbrc.2001.4998 11396950

[111] He H, Li W, Wu D, Nagy R, Liyanarachchi S, Akagi K, Jendrzejewski J, Jiao H, Hoag K, Wen B, Srinivas M, Waidyaratne G, Wang R, Wojcicka A, Lattimer IR, Stachlewska E, Czetwertynska M, Dlugosinska J, Gierlikowski W, Ploski R, Krawczyk M, Jazdzewski K, Kere J, Symer DE, Jin V, Wang Q, de la Chapelle A (2013). Ultra-rare mutation in long-range enhancer predisposes to thyroid carcinoma with high penetrance. PLoS One..

[112] Xing M (2005). The T1799A BRAF mutation is not a germline mutation in familial nonmedullary thyroid cancer. Clin Endocrinol (Oxf)..

[113] LiVolsi VA, Baraban E, Baloch ZW (2017). Familial thyroid carcinoma: the road less traveled in thyroid pathology – an update. Diagnostic Histopathology..

[114] LiVolsi V, Eng C, Foulkes WD, Nosé V, Schmid KW, Lloyd RV, Osamura RY, Klöppel G, Rosai J (2017). Familial non-medullary thyroid cancer. WHO Classification of Tumours of Endocrine Organs.

[115] Guilmette J, Nosé V (2018). Hereditary and familial thyroid tumours. Histopathology..

[116] Tan MH, Mester J, Peterson C, Yang Y, Chen JL, Rybicki LA, Milas K, Pederson H, Remzi B, Orloff MS, Eng C (2011). A clinical scoring system for selection of patients for PTEN mutation testing is proposed on the basis of a prospective study of 3042 probands. Am J Hum Genet..

[117] Khan NE, Bauer AJ, Doros L, Schultz KA, Decastro RM, Harney LA, Kase RG, Carr AG, Harris AK, Williams GM, Dehner LP, Messinger YH, Stewart DR (2017). Macrocephaly associated with the DICER1 syndrome. Genet Med..

[118] Perakakis N, Flohr F, Kayser G, Thomusch O, Parsons L, Billmann F, von Dobschuetz E, Rondot S, Seufert J, Laubner K (2016). Multiple endocrine neoplasia type 1 associated with a new germline Men1 mutation in a family with atypical tumor phenotype. Hormones (Athens)..

[119] Winer DA, Winer S, Rotstein L, Asa SL, Mete O (2012). Villous papillary thyroid carcinoma: a variant associated with marfan syndrome. Endocr Pathol..

[120] Wolf KI, Jacobs MF, Mehra R, Begani P, Davenport MS, Marentette LJ, Basura GJ, Hughes DT, Else T (2019). A Family With a Carotid Body Paraganglioma and Thyroid Neoplasias With a New SDHAF2 Germline Variant. J Endocr Soc..

[121] Kösem M, Kotan C, Algün E, Topal C (2002). Simultaneous occurrence of papillary intrafollicular and microcarcinomas with bilateral medullary microcarcinoma of the thyroid in a patient with multiple endocrine neoplasia type 2A: report of a case. Surg Today..

[122] Giacomelli L, Guerriero G, Falvo L, Altomare V, Chiesa C, Ferri S, Stio F. Simultaneous occurrence of medullary carcinoma and papillary microcarcinoma of thyroid in a patient with MEN 2A syndrome. report of a case. Tumori. 2007;93(1):109–11. 10.1177/030089160709300121 17455883

[123] Harach HR, Soubeyran I, Brown A, Bonneau D, Longy M (1999). Thyroid pathologic findings in patients with Cowden disease. Ann Diagn Pathol..

[124] Cameselle-Teijeiro J (2010). The pathologist's role in familial nonmedullary thyroid tumors. Int J Surg Pathol..

[125] Cameselle-Teijeiro J, Fachal C, Cabezas-Agrícola JM, Alfonsín-Barreiro N, Abdulkader I, Vega-Gliemmo A, Hermo JA (2015). Thyroid Pathology Findings in Cowden Syndrome: A Clue for the Diagnosis of the PTEN Hamartoma Tumor Syndrome. Am J Clin Pathol..

[126] Arends MJ, Carneiro F, Lax SF, Lazar AJ, editors. Genetic tumour syndromes of the digestive system. In: WHO Classification of Tumours Editorial Board. Digestive system tumours. 5th ed. Lyon: IARC. 2019; pp 511–50.

[127] Vasen HF, Möslein G, Alonso A, Aretz S, Bernstein I, Bertario L, Blanco I, Bülow S, Burn J, Capella G, Colas C, Engel C, Frayling I, Friedl W, Hes FJ, Hodgson S, Järvinen H, Mecklin JP, Møller P, Myrhøi T, Nagengast FM, Parc Y, Phillips R, Clark SK, de Leon MP, Renkonen-Sinisalo L, Sampson JR, Stormorken A, Tejpar S, Thomas HJ, Wijnen J (2008). Guidelines for the clinical management of familial adenomatous polyposis (FAP). Gut..

[128] Knudsen AL, Bülow S, Tomlinson I, Möslein G, Heinimann K, Christensen IJ; AFAP Study Group. Attenuated familial adenomatous polyposis: results from an international collaborative study. Colorectal Dis. 2010;12(10 Online):e243–9. 10.1111/j.1463-1318.2010.02218.x 20105204

[129] Rehan S, Aye K (2020). In patients with a positive family history of familial adenomatous polyposis can the condition be diagnosed from the presence of congenital hypertrophy of the retinal pigment epithelium detected via an eye examination: A systematic review. Clin Exp Ophthalmol..

[130] Saito Y, Hinoi T, Ueno H, Kobayashi H, Konishi T, Ishida F, Yamaguchi T, Inoue Y, Kanemitsu Y, Tomita N, Matsubara N, Komori K, Kotake K, Nagasaka T, Hasegawa H, Koyama M, Ohdan H, Watanabe T, Sugihara K, Ishida H (2016). Risk Factors for the Development of Desmoid Tumor After Colectomy in Patients with Familial Adenomatous Polyposis: Multicenter Retrospective Cohort Study in Japan. Ann Surg Oncol..

[131] Li J, Woods SL, Healey S, Beesley J, Chen X, Lee JS, Sivakumaran H, Wayte N, Nones K, Waterfall JJ, Pearson J, Patch AM, Senz J, Ferreira MA, Kaurah P, Mackenzie R, Heravi-Moussavi A, Hansford S, Lannagan TRM, Spurdle AB, Simpson PT, da Silva L, Lakhani SR, Clouston AD, Bettington M, Grimpen F, Busuttil RA, Di Costanzo N, Boussioutas A, Jeanjean M, Chong G, Fabre A, Olschwang S, Faulkner GJ, Bellos E, Coin L, Rioux K, Bathe OF, Wen X, Martin HC, Neklason DW, Davis SR, Walker RL, Calzone KA, Avital I, Heller T, Koh C, Pineda M, Rudloff U, Quezado M, Pichurin PN, Hulick PJ, Weissman SM, Newlin A, Rubinstein WS, Sampson JE, Hamman K, Goldgar D, Poplawski N, Phillips K, Schofield L, Armstrong J, Kiraly-Borri C, Suthers GK, Huntsman DG, Foulkes WD, Carneiro F, Lindor NM, Edwards SL, French JD, Waddell N, Meltzer PS, Worthley DL, Schrader KA, Chenevix-Trench G (2016). Point Mutations in Exon 1B of APC Reveal Gastric Adenocarcinoma and Proximal Polyposis of the Stomach as a Familial Adenomatous Polyposis Variant. Am J Hum Genet..

[132] Groves C, Lamlum H, Crabtree M, Williamson J, Taylor C, Bass S, Cuthbert-Heavens D, Hodgson S, Phillips R, Tomlinson I (2002). Mutation cluster region, association between germline and somatic mutations and genotype-phenotype correlation in upper gastrointestinal familial adenomatous polyposis. Am J Pathol..

[133] Kennedy RD, Potter DD, Moir CR, El-Youssef M (2014). The natural history of familial adenomatous polyposis syndrome: a 24 year review of a single center experience in screening, diagnosis, and outcomes. J Pediatr Surg..

[134] Cameselle-Teijeiro JM, Peteiro-González D, Caneiro-Gómez J, Sánchez-Ares M, Abdulkader I, Eloy C, Melo M, Amendoeira I, Soares P, Sobrinho-Simões M (2018). Cribriform-morular variant of thyroid carcinoma: a neoplasm with distinctive phenotype associated with the activation of the WNT/β-catenin pathway. Mod Pathol..

[135] Cameselle-Teijeiro JM, Sobrinho-Simões M (2019). Cribriform-morular variant of thyroid carcinoma. Pathologica..

[136] Uchino S, Ishikawa H, Miyauchi A, Hirokawa M, Noguchi S, Ushiama M, Yoshida T, Michikura M, Sugano K, Sakai T (2016). Age- and Gender-Specific Risk of Thyroid Cancer in Patients With Familial Adenomatous Polyposis. J Clin Endocrinol Metab..

[137] Park J, Kim JW, Park H, Park SY, Kim TH, Kim SW, Oh YL, Chung JH (2019). Multifocality in a Patient with Cribriform-Morular Variant of Papillary Thyroid Carcinoma Is an Important Clue for the Diagnosis of Familial Adenomatous Polyposis. Thyroid..

[138] Nieminen TT, Walker CJ, Olkinuora A, Genutis LK, O'Malley M, Wakely PE, LaGuardia L, Koskenvuo L, Arola J, Lepistö AH, Brock P, Yilmaz AS, Eisfeld AK, Church JM, Peltomäki P, de la Chapelle A (2020). Thyroid Carcinomas That Occur in Familial Adenomatous Polyposis Patients Recurrently Harbor Somatic Variants in APC, BRAF, and KTM2D. Thyroid..

[139] Crail HW (1949). Multiple primary malignancies arising in the rectum, brain, and thyroid; report of a case. U S Nav Med Bull..

[140] Harach HR, Williams GT, Williams ED (1994). Familial adenomatous polyposis associated thyroid carcinoma: a distinct type of follicular cell neoplasm. Histopathology..

[141] Cameselle-Teijeiro J, Chan JK (1999). Cribriform-morular variant of papillary carcinoma: a distinctive variant representing the sporadic counterpart of familial adenomatous polyposis-associated thyroid carcinoma?. Mod Pathol..

[142] Baloch ZW, Segal JP, Livolsi VA (2011). Unique growth pattern in papillary carcinoma of the thyroid gland mimicking adenoid cystic carcinoma. Endocr Pathol..

[143] Nakazawa T, Celestino R, Machado JC, Cameselle-Teijeiro JM, Vinagre J, Eloy C, Benserai F, Lameche S, Soares P, Sobrinho-Simões M (2013). Cribriform-morular variant of papillary thyroid carcinoma displaying poorly differentiated features. Int J Surg Pathol..

[144] Mohindra S, Sakr H, Sturgis C, Chute DJ (2018). LEF-1 is a Sensitive Marker of Cribriform Morular Variant of Papillary Thyroid Carcinoma. Head Neck Pathol..

[145] Cameselle-Teijeiro J, Alberte-Lista L, Peteiro-González D, Abdulkader-Nallib I, Reyes-Santías R, Soares P, Sobrinho-Simões M (2012). CDX2 Expression in Some Variants of Papillary Thyroid Carcinoma. Am J Clin Pathol..

[146] Sakurai K, Onouchi T, Yamada S, Baba Y, Murata T, Tsukamoto T, Kuroda M, Urano M (2019). Cytohistology of morule in cribriform-morular variant of papillary thyroid carcinoma. Malays J Pathol..

[147] Cameselle-Teijeiro JM, Eloy C, Sobrinho-Simões M (2020). Pitfalls in Challenging Thyroid Tumors: Emphasis on Differential Diagnosis and Ancillary Biomarkers. Endocr Pathol..

[148] Laforga JB, Pedro T, Gasent JM (2020). Pulmonary metastasis of cribriform-morular variant of thyroid carcinoma mimicking primary adenocarcinoma of the lung: A potential pitfall. Diagn Cytopathol..

[149] Enomoto K, Tamagawa S, Kumashiro N, Warigaya K, Takeda S, Gunduz M, Murata SI, Hotomi M (2020). A rare case of the recurrent surgery for cribriform-morular variant of papillary thyroid carcinoma. Int J Surg Case Rep..

[150] Cameselle-Teijeiro J, Menasce LP, Yap BK, Colaco RJ, Castro P, Celestino R, Ruíz-Ponte C, Soares P, Sobrinho-Simões M (2009). Cribriform-morular variant of papillary thyroid carcinoma: molecular characterization of a case with neuroendocrine differentiation and aggressive behavior. Am J Clin Pathol..

[151] Oh EJ, Lee S, Bae JS, Kim Y, Jeon S, Jung CK (2017). TERT Promoter Mutation in an Aggressive Cribriform Morular Variant of Papillary Thyroid Carcinoma. Endocr Pathol..

[152] Tsuji H, Yasuoka H, Nakamura Y, Hirokawa M, Hiroshima T, Sakamaki Y, Miyauchi A, Tsujimoto M (2018). Aggressive cribriform-morular variant of papillary thyroid carcinoma: Report of an unusual case with pulmonary metastasis displaying poorly differentiated features. Pathol Int..

[153] Ito Y, Ishikawa H, Kihara M, Hirokawa M, Kiyota N, Kasahara T, Miyauchi A (2019). Control of Lung Metastases and Colon Polyposis with Lenvatinib Therapy in a Patient with Cribriform-Morular Variant of Papillary Thyroid Carcinoma and an APC Gene Mutation: A Case Study. Thyroid..

[154] Hirokawa M, Matsuda K, Kudo T, Higuchi M, Suzuki A, Takada N, Nakashima M, Miyauchi A (2019). Cribriform-Morular Variant of Papillary Thyroid Carcinoma Shows High Ki-67 Labeling Indices, despite Its Excellent Prognosis. Pathobiology..

[155] Lamlum H, Ilyas M, Rowan A, Clark S, Johnson V, Bell J, Frayling I, Efstathiou J, Pack K, Payne S, Roylance R, Gorman P, Sheer D, Neale K, Phillips R, Talbot I, Bodmer W, Tomlinson I (1999). The type of somatic mutation at APC in familial adenomatous polyposis is determined by the site of the germline mutation: a new facet to Knudson's 'two-hit' hypothesis. Nat Med..

[156] Truta B, Allen BA, Conrad PG, Kim YS, Berk T, Gallinger S, Bapat B, Terdiman JP, Sleisenger MH (2003). Genotype and phenotype of patients with both familial adenomatous polyposis and thyroid carcinoma. Fam Cancer..

[157] Uchino S, Noguchi S, Yamashita H, Yamashita H, Watanabe S, Ogawa T, Tsuno A, Murakami A, Miyauchi A (2006). Mutational analysis of the APC gene in cribriform-morula variant of papillary thyroid carcinoma. World J Surg..

[158] Cetta F, Montalto G, Gori M, Curia MC, Cama A, Olschwang S (2000). Germline mutations of the APC gene in patients with familial adenomatous polyposis-associated thyroid carcinoma: results from a European cooperative study. J Clin Endocrinol Metab..

[159] Septer S, Lawson CE, Anant S, Attard T (2016). Familial adenomatous polyposis in pediatrics: natural history, emerging surveillance and management protocols, chemopreventive strategies, and areas of ongoing debate. Fam Cancer..

[160] Yang K, Wang X, Zhang H, Wang Z, Nan G, Li Y, Zhang F, Mohammed MK, Haydon RC, Luu HH, Bi Y, He TC (2016). The evolving roles of canonical WNT signaling in stem cells and tumorigenesis: implications in targeted cancer therapies. Lab Invest..

[161] Miyaki M, Iijima T, Ishii R, Hishima T, Mori T, Yoshinaga K, Takami H, Kuroki T, Iwama T (2000). Molecular evidence for multicentric development of thyroid carcinomas in patients with familial adenomatous polyposis. Am J Pathol..

[162] Cameselle-Teijeiro J, Ruiz-Ponte C, Loidi L, Suarez-Peñaranda J, Baltar J, Sobrinho-Simoes M (2001). Somatic but not germline mutation of the APC gene in a case of cribriform-morular variant of papillary thyroid carcinoma. Am J Clin Pathol..

[163] Aydemirli MD, van der Tuin K, Hes FJ, van den Ouweland AMW, van Wezel T, Kapiteijn E, Morreau H (2020). A unique case of two somatic APC mutations in an early onset cribriform-morular variant of papillary thyroid carcinoma and overview of the literature. Fam Cancer..

[164] Xu B, Yoshimoto K, Miyauchi A, Kuma S, Mizusawa N, Hirokawa M, Sano T (2003). Cribriform-morular variant of papillary thyroid carcinoma: a pathological and molecular genetic study with evidence of frequent somatic mutations in exon 3 of the beta-catenin gene. J Pathol..

[165] Cameselle-Teijeiro JM, Peteiro-González D, Carreira M, Abdulkader I, Reyes-Santías R, Celestino R, Romero Rojas C, Ruíz Ponte C, Soares P, Casanueva F, Sobrinho-Simões. Molecular alterations in the cribriform-morular variant of papillary thyroid carcinoma. Virchows Arch. 2016;469(Suppl 1):S72.

[166] Peteiro González Diego. Clinical and molecular study of the cribriform-morular variant of papillary thyroid carcinoma. Doctoral Thesis. University of Santiago de Compostela. Spain. 2016 (in Spanish).

[167] Cetta F, Toti P, Petracci M, Montalto G, Disanto A, Lorè F, Fusco A (1997). Thyroid carcinoma associated with familial adenomatous polyposis. Histopathology..

[168] Soravia C, Sugg SL, Berk T, Mitri A, Cheng H, Gallinger S, Cohen Z, Asa SL, Bapat BV (1999). Familial adenomatous polyposis-associated thyroid cancer: a clinical, pathological, and molecular genetics study. Am J Pathol..

[169] Cetta F, Curia MC, Montalto G, Gori M, Cama A, Battista P, Barbarisi A (2001). Thyroid carcinoma usually occurs in patients with familial adenomatous polyposis in the absence of biallelic inactivation of the adenomatous polyposis coli gene. J Clin Endocrinol Metab..

[170] Giannelli SM, McPhaul L, Nakamoto J, Gianoukakis AG (2014). Familial adenomatous polyposis-associated, cribriform morular variant of papillary thyroid carcinoma harboring a K-RAS mutation: case presentation and review of molecular mechanisms. Thyroid..

[171] Kwon MJ, Rho YS, Jeong JC, Shin HS, Lee JS, Cho SJ, Nam ES (2015). Cribriform-morular variant of papillary thyroid carcinoma: a study of 3 cases featuring the PIK3CA mutation. Hum Pathol..

[172] Schuetze D, Hoschar AP, Seethala RR, Assaad A, Zhang X, Hunt JL (2009). The T1799A BRAF mutation is absent in cribriform-morular variant of papillary carcinoma. Arch Pathol Lab Med..

[173] Eng C. PTEN Hamartoma Tumor Syndrome. 2001 Nov 29 [updated 2016 Jun 2]. In: Adam MP, Ardinger HH, Pagon RA, Wallace SE, Bean LJH, Stephens K, Amemiya A, editors. GeneReviews® [Internet]. Seattle (WA): University of Washington, Seattle; 1993–2020. PMID: 20301661.

[174] Macken WL, Tischkowitz M, Lachlan KL (2019). PTEN Hamartoma tumor syndrome in childhood: A review of the clinical literature. Am J Med Genet C Semin Med Genet..

[175] Yehia L, Ngeow J, Eng C (2019). PTEN-opathies: from biological insights to evidence-based precision medicine. J Clin Invest..

[176] Jonker LA, Lebbink CA, Jongmans MCJ, Nievelstein RAJ, Merks JHM, Nieveen van Dijkum EJM, Links TP, Hoogerbrugge N, van Trotsenburg ASP, van Santen HM. Recommendations on Surveillance for Differentiated Thyroid Carcinoma in Children with PTEN Hamartoma Tumor Syndrome. Eur Thyroid J. 2020;9(5):234–42. 10.1159/000508872 PMC7548843 33088791

[177] Tischkowitz M, Colas C, Pouwels S, Hoogerbrugge N; PHTS Guideline Development Group; European Reference Network GENTURIS. Cancer Surveillance Guideline for individuals with PTEN hamartoma tumour syndrome. Eur J Hum Genet. 2020;28(10):1387–93. 10.1038/s41431-020-0651-7 PMC7608293 32533092

[178] Kurek KC, Howard E, Tennant LB, Upton J, Alomari AI, Burrows PE, Chalache K, Harris DJ, Trenor CC, Eng C, Fishman SJ, Mulliken JB, Perez-Atayde AR, Kozakewich HP (2012). PTEN hamartoma of soft tissue: a distinctive lesion in PTEN syndromes. Am J Surg Pathol..

[179] Bennett KL, Mester J, Eng C (2010). Germline epigenetic regulation of KILLIN in Cowden and Cowden-like syndrome. JAMA..

[180] Ngeow J, Mester J, Rybicki LA, Ni Y, Milas M, Eng C (2011). Incidence and clinical characteristics of thyroid cancer in prospective series of individuals with Cowden and Cowden-like syndrome characterized by germline PTEN, SDH, or KLLN alterations. J Clin Endocrinol Metab..

[181] Orloff MS, He X, Peterson C, Chen F, Chen JL, Mester JL, Eng C (2013). Germline PIK3CA and AKT1 mutations in Cowden and Cowden-like syndromes. Am J Hum Genet..

[182] Tian W, Huang Y, Sun L, Guo Y, Zhao S, Lin M, Dong X, Zhong W, Yin Y, Chen Z, Zhang N, Zhang Y, Wang L, Lin J, Yan Z, Yang X, Zhao J, Qiu G, Zhang J, Wu Z, Wu N; (Deciphering Disorders Involving Scoliosis, COmorbidities) study group. Phenotypic and genetic spectrum of isolated macrodactyly: somatic mosaicism of PIK3CA and AKT1 oncogenic variants. Orphanet J Rare Dis. 2020;15(1):288. 10.1186/s13023-020-01572-9 PMC7556951 33054853

[183] Riegert-Johnson DL, Gleeson FC, Roberts M, Tholen K, Youngborg L, Bullock M, Boardman LA (2010). Cancer and Lhermitte-Duclos disease are common in Cowden syndrome patients. Hered Cancer Clin Pract..

[184] Tan MH, Mester JL, Ngeow J, Rybicki LA, Orloff MS, Eng C (2012). Lifetime cancer risks in individuals with germline PTEN mutations. Clin Cancer Res..

[185] Milas M, Mester J, Metzger R, Shin J, Mitchell J, Berber E, Siperstein AE, Eng C (2012). Should patients with Cowden syndrome undergo prophylactic thyroidectomy?. Surgery..

[186] Bubien V, Bonnet F, Brouste V, Hoppe S, Barouk-Simonet E, David A, Edery P, Bottani A, Layet V, Caron O, Gilbert-Dussardier B, Delnatte C, Dugast C, Fricker JP, Bonneau D, Sevenet N, Longy M, Caux F; French Cowden Disease Network. High cumulative risks of cancer in patients with PTEN hamartoma tumour syndrome. J Med Genet. 2013;50(4):255–63. 10.1136/jmedgenet-2012-101339 23335809

[187] Szabo Yamashita T, Baky FJ, McKenzie TJ, Thompson GB, Farley DR, Lyden ML, Dy BM (2020). Occurrence and Natural History of Thyroid Cancer in Patients with Cowden Syndrome. Eur Thyroid J..

[188] Barletta JA, Bellizzi AM, Hornick JL (2011). Immunohistochemical staining of thyroidectomy specimens for PTEN can aid in the identification of patients with Cowden syndrome. Am J Surg Pathol..

[189] Smith JR, Marqusee E, Webb S, Nose V, Fishman SJ, Shamberger RC, Frates MC, Huang SA (2011). Thyroid nodules and cancer in children with PTEN hamartoma tumor syndrome. J Clin Endocrinol Metab..

[190] Harach HR (2001). Familial nonmedullary thyroid neoplasia. Endocr Pathol..

[191] Lloyd KM 2nd, Dennis M. Cowden's disease. A possible new symptom complex with multiple system involvement. Ann Intern Med. 1963;58:136–42. 10.7326/0003-4819-58-1-136 13931122

[192] Laury AR, Bongiovanni M, Tille JC, Kozakewich H, Nosé V (2011). Thyroid pathology in PTEN-hamartoma tumor syndrome: characteristic findings of a distinct entity. Thyroid..

[193] Peiretti V, Mussa A, Feyles F, Tuli G, Santanera A, Molinatto C, Ferrero GB, Corrias A (2013). Thyroid involvement in two patients with Bannayan-Riley-Ruvalcaba syndrome. J Clin Res Pediatr Endocrinol..

[194] Zambrano E, Holm I, Glickman J, Huang S, Perez-Atayde A, Kozakewich HP, Shamberger RC, Nosé V (2004). Abnormal distribution and hyperplasia of thyroid C-cells in PTEN-associated tumor syndromes. Endocr Pathol..

[195] Hall JE, Abdollahian DJ, Sinard RJ (2013). Thyroid disease associated with Cowden syndrome: A meta-analysis. Head Neck..

[196] Nosé V (2016). Genodermatosis Affecting the Skin and Mucosa of the Head and Neck: Clinicopathologic, Genetic, and Molecular Aspect–PTEN-Hamartoma Tumor Syndrome/Cowden Syndrome. Head Neck Pathol..

[197] Inoki K, Li Y, Zhu T, Wu J, Guan KL (2002). TSC2 is phosphorylated and inhibited by Akt and suppresses mTOR signalling. Nat Cell Biol..

[198] Lee YR, Chen M, Pandolfi PP. The functions and regulation of the PTEN tumour suppressor: new modes and prospects. Nat Rev Mol Cell Biol. 2018;19(9):547–562. 10.1038/s41580-018-0015-0 29858604

[199] Zhou XP, Waite KA, Pilarski R, Hampel H, Fernandez MJ, Bos C, Dasouki M, Feldman GL, Greenberg LA, Ivanovich J, Matloff E, Patterson A, Pierpont ME, Russo D, Nassif NT, Eng C (2003). Germline PTEN promoter mutations and deletions in Cowden/Bannayan-Riley-Ruvalcaba syndrome result in aberrant PTEN protein and dysregulation of the phosphoinositol-3-kinase/Akt pathway. Am J Hum Genet..

[200] Teresi RE, Zbuk KM, Pezzolesi MG, Waite KA, Eng C (2007). Cowden syndrome-affected patients with PTEN promoter mutations demonstrate abnormal protein translation. Am J Hum Genet..

[201] Ni Y, He X, Chen J, Moline J, Mester J, Orloff MS, Ringel MD, Eng C (2012). Germline SDHx variants modify breast and thyroid cancer risks in Cowden and Cowden-like syndrome via FAD/NAD-dependant destabilization of p53. Hum Mol Genet..

[202] Yehia L, Eng C (2018). 65 years of the double helix: One gene, many endocrine and metabolic syndromes: PTEN-opathies and precision medicine. Endocr Relat Cancer..

[203] Colby S, Yehia L, Niazi F, Chen J, Ni Y, Mester JL, Eng C (2016). Exome sequencing reveals germline gain-of-function EGFR mutation in an adult with Lhermitte-Duclos disease. Cold Spring Harb Mol Case Stud..

[204] Ngeow J, Ni Y, Tohme R, Song Chen F, Bebek G, Eng C (2014). Germline alterations in RASAL1 in Cowden syndrome patients presenting with follicular thyroid cancer and in individuals with apparently sporadic epithelial thyroid cancer. J Clin Endocrinol Metab..

[205] Pradella LM, Zuntini R, Magini P, Ceccarelli C, Neri I, Cerasoli S, Graziano C, Gasparre G, Turchetti D (2011). Two distinct thyroid tumours in a patient with Cowden syndrome carrying both a 10q23 and a mitochondrial DNA germline deletion. J Med Genet..

[206] Foulkes WD, Kovacs K, Lloyd RV, Osamura RY, Klöppel G, Rosai J (2017). DICER1 syndrome. WHO Classification of Tumours of Endocrine Organs.

[207] Solarski M, Rotondo F, Foulkes WD, Priest JR, Syro LV, Butz H, Cusimano MD, Kovacs K (2018). DICER1 gene mutations in endocrine tumors. Endocr Relat Cancer..

[208] Schultz KAP, Williams GM, Kamihara J, Stewart DR, Harris AK, Bauer AJ, Turner J, Shah R, Schneider K, Schneider KW, Carr AG, Harney LA, Baldinger S, Frazier AL, Orbach D, Schneider DT, Malkin D, Dehner LP, Messinger YH, Hill DA (2018). DICER1 and Associated Conditions: Identification of At-risk Individuals and Recommended Surveillance Strategies. Clin Cancer Res..

[209] Schultz KAP, Stewart DR, Kamihara J, et al. DICER1 Tumor Predisposition. 2014 Apr 24 [Updated 2020 Apr 30]. In: Adam MP, Ardinger HH, Pagon RA, et al., editors. GeneReviews® [Internet]. Seattle (WA): University of Washington, Seattle; 1993–2020. Available from: https://www.ncbi.nlm.nih.gov/books/NBK196157/

[210] Foulkes WD, Priest JR, Duchaine TF (2014). DICER1: mutations, microRNAs and mechanisms. Nat Rev Cancer..

[211] de Kock L, Sabbaghian N, Plourde F, Srivastava A, Weber E, Bouron-Dal Soglio D, Hamel N, Choi JH, Park SH, Deal CL, Kelsey MM, Dishop MK, Esbenshade A, Kuttesch JF, Jacques TS, Perry A, Leichter H, Maeder P, Brundler MA, Warner J, Neal J, Zacharin M, Korbonits M, Cole T, Traunecker H, McLean TW, Rotondo F, Lepage P, Albrecht S, Horvath E, Kovacs K, Priest JR, Foulkes WD (2014). Pituitary blastoma: a pathognomonic feature of germ-line DICER1 mutations. Acta Neuropathol..

[212] Darrat I, Bedoyan JK, Chen M, Schuette JL, Lesperance MM (2013). Novel DICER1 mutation as cause of multinodular goiter in children. Head Neck..

[213] Oue T, Inoue M, Kubota A, Kuwae Y, Kawa K (2008). Pediatric thyroid cancer arising after treatment for pleuropulmonary blastoma. Pediatr Blood Cancer..

[214] Rome A, Gentet JC, Coze C, André N (2008). Pediatric thyroid cancer arising as a fourth cancer in a child with pleuropulmonary blastoma. Pediatr Blood Cancer..

[215] de Kock L, Sabbaghian N, Soglio DB, Guillerman RP, Park BK, Chami R, Deal CL, Priest JR, Foulkes WD (2014). Exploring the association between DICER1 mutations and differentiated thyroid carcinoma. J Clin Endocrinol Metab..

[216] Rutter MM, Jha P, Schultz KA, Sheil A, Harris AK, Bauer AJ, Field AL, Geller J, Hill DA (2016). DICER1 Mutations and Differentiated Thyroid Carcinoma: Evidence of a Direct Association. J Clin Endocrinol Metab..

[217] Chi M, Gilman AD, Iroegbu N (2011). Management of metastatic ovarian Sertoli-Leydig cell tumor with sporadic multinodular goiter: a case report and literature review. Future Oncol..

[218] Rio Frio T, Bahubeshi A, Kanellopoulou C, Hamel N, Niedziela M, Sabbaghian N, Pouchet C, Gilbert L, O'Brien PK, Serfas K, Broderick P, Houlston RS, Lesueur F, Bonora E, Muljo S, Schimke RN, Bouron-Dal Soglio D, Arseneau J, Schultz KA, Priest JR, Nguyen VH, Harach HR, Livingston DM, Foulkes WD, Tischkowitz M (2011). DICER1 mutations in familial multinodular goiter with and without ovarian Sertoli-Leydig cell tumors. JAMA..

[219] Rossing M, Gerdes AM, Juul A, Rechnitzer C, Rudnicki M, Nielsen FC, Vo Hansen T (2014). A novel DICER1 mutation identified in a female with ovarian Sertoli-Leydig cell tumor and multinodular goiter: a case report. J Med Case Rep..

[220] Wu Y, Chen D, Li Y, Bian L, Ma T, Xie M (2014). DICER1 mutations in a patient with an ovarian Sertoli-Leydig tumor, well-differentiated fetal adenocarcinoma of the lung, and familial multinodular goiter. Eur J Med Genet..

[221] Apellaniz-Ruiz M, de Kock L, Sabbaghian N, Guaraldi F, Ghizzoni L, Beccuti G, Foulkes WD (2018). Familial multinodular goiter and Sertoli-Leydig cell tumors associated with a large intragenic in-frame DICER1 deletion. Eur J Endocrinol..

[222] Haley M, Bindal P, McAuliffe A, Vredenburgh J (2019). A family with Sertoli-Leydig cell tumour, multinodular goiter, and DICER1 mutation. Curr Oncol..

[223] Durieux E, Descotes F, Mauduit C, Decaussin M, Guyetant S, Devouassoux-Shisheboran M (2016). The co-occurrence of an ovarian Sertoli-Leydig cell tumor with a thyroid carcinoma is highly suggestive of a DICER1 syndrome. Virchows Arch..

[224] Chernock RD, Rivera B, Borrelli N, Hill DA, Fahiminiya S, Shah T, Chong AS, Aqil B, Mehrad M, Giordano TJ, Sheridan R, Rutter MM, Dehner LP, Foulkes WD, Nikiforov YE (2020). Poorly differentiated thyroid carcinoma of childhood and adolescence: a distinct entity characterized by DICER1 mutations. Mod Pathol..

[225] Wasserman JD, Sabbaghian N, Fahiminiya S, Chami R, Mete O, Acker M, Wu MK, Shlien A, de Kock L, Foulkes WD (2018). DICER1 Mutations Are Frequent in Adolescent-Onset Papillary Thyroid Carcinoma. J Clin Endocrinol Metab..

[226] Ravella L, Lopez J, Descotes F, Lifante JC, David C, Decaussin-Petrucci M. Carcinome papillaire thyroïdien variante solide/trabéculaire avec mutation DICER1 chez une enfant de 11 ans [DICER1 mutated, solid/trabecular thyroid papillary carcinoma in an 11-year-old child]. Ann Pathol. 2018;38(5):316–20. French. 10.1016/j.annpat.2018.04.003 29884466

[227] Agaimy A, Witkowski L, Stoehr R, Cuenca JCC, González-Muller CA, Brütting A, Bährle M, Mantsopoulos K, Amin RMS, Hartmann A, Metzler M, Amr SS, Foulkes WD, Sobrinho-Simões M, Eloy C. Malignant teratoid tumor of the thyroid gland: an aggressive primitive multiphenotypic malignancy showing organotypical elements and frequent DICER1 alterations-is the term "thyroblastoma" more appropriate? Virchows Arch. 2020 Jun 7. 10.1007/s00428-020-02853-1. Epub ahead of print. PMID: 32507920. 10.1007/s00428-020-02853-1 PMC7683491 32507920

[228] Rabinowits G, Barletta J, Sholl LM, Reche E, Lorch J, Goguen L (2017). Successful Management of a Patient with Malignant Thyroid Teratoma. Thyroid..

[229] Yang J, Sarita-Reyes C, Kindelberger D, Zhao Q (2018). A rare malignant thyroid carcinosarcoma with aggressive behavior and DICER1 gene mutation: a case report with literature review. Thyroid Res..

[230] Rooper LM, Bynum JP, Miller KP, Lin MT, Gagan J, Thompson LDR, Bishop JA (2020). Recurrent DICER1 Hotspot Mutations in Malignant Thyroid Gland Teratomas: Molecular Characterization and Proposal for a Separate Classification. Am J Surg Pathol..

[231] Hill DA, Ivanovich J, Priest JR, Gurnett CA, Dehner LP, Desruisseau D, Jarzembowski JA, Wikenheiser-Brokamp KA, Suarez BK, Whelan AJ, Williams G, Bracamontes D, Messinger Y, Goodfellow PJ (2009). DICER1 mutations in familial pleuropulmonary blastoma. Science..

[232] Pugh TJ, Yu W, Yang J, Field AL, Ambrogio L, Carter SL, Cibulskis K, Giannikopoulos P, Kiezun A, Kim J, McKenna A, Nickerson E, Getz G, Hoffher S, Messinger YH, Dehner LP, Roberts CW, Rodriguez-Galindo C, Williams GM, Rossi CT, Meyerson M, Hill DA (2014). Exome sequencing of pleuropulmonary blastoma reveals frequent biallelic loss of TP53 and two hits in DICER1 resulting in retention of 5p-derived miRNA hairpin loop sequences. Oncogene..

[233] Herriges JC, Brown S, Longhurst M, Ozmore J, Moeschler JB, Janze A, Meck J, South ST, Andersen EF (2019). Identification of two 14q32 deletions involving DICER1 associated with the development of DICER1-related tumors. Eur J Med Genet..

[234] Cancer Genome Atlas Research Network (2014). Integrated genomic characterization of papillary thyroid carcinoma. Cell..

[235] Bongiovanni M, Sykiotis GP, La Rosa S, Bisig B, Trimech M, Missiaglia E, Gremaud M, Salvatori Chappuis V, De Vito C, Sciarra A, Foulkes WD, Pusztaszeri M (2020). Macrofollicular Variant of Follicular Thyroid Carcinoma: A Rare Underappreciated Pitfall in the Diagnosis of Thyroid Carcinoma. Thyroid..

[236] Gullo I, Batista R, Rodrigues-Pereira P, Soares P, Barroca H, do Bom-Sucesso M, Sobrinho-Simões M. Multinodular Goiter Progression Toward Malignancy in a Case of DICER1 Syndrome: Histologic and Molecular Alterations. Am J Clin Pathol. 2018;149(5):379–86. 10.1093/ajcp/aqy004 29538609

[237] Carney JA, Gordon H, Carpenter PC, Shenoy BV, Go VL (1985). The complex of myxomas, spotty pigmentation, and endocrine overactivity. Medicine (Baltimore)..

[238] Carney JA, Hruska LS, Beauchamp GD, Gordon H (1986). Dominant inheritance of the complex of myxomas, spotty pigmentation, and endocrine overactivity. Mayo Clin Proc..

[239] Carney JA. Psammomatous melanotic schwannoma. A distinctive, heritable tumor with special associations, including cardiac myxoma and the Cushing syndrome. Am J Surg Pathol. 1990;14(3):206–22. 2305928

[240] Carney JA, Ferreiro JA. The epithelioid blue nevus. A multicentric familial tumor with important associations, including cardiac myxoma and psammomatous melanotic schwannoma. Am J Surg Pathol. 1996;20(3):259–72. 10.1097/00000478-199603000-00001 8772778

[241] Stratakis CA, Courcoutsakis NA, Abati A, Filie A, Doppman JL, Carney JA, Shawker T (1997). Thyroid gland abnormalities in patients with the syndrome of spotty skin pigmentation, myxomas, endocrine overactivity, and schwannomas (Carney complex). J Clin Endocrinol Metab..

[242] Carney JA, Boccon-Gibod L, Jarka DE, Tanaka Y, Swee RG, Unni KK, Stratakis CA (2001). Osteochondromyxoma of bone: a congenital tumor associated with lentigines and other unusual disorders. Am J Surg Pathol..

[243] Bertherat J, Horvath A, Groussin L, Grabar S, Boikos S, Cazabat L, Libe R, René-Corail F, Stergiopoulos S, Bourdeau I, Bei T, Clauser E, Calender A, Kirschner LS, Bertagna X, Carney JA, Stratakis CA (2009). Mutations in regulatory subunit type 1A of cyclic adenosine 5'-monophosphate-dependent protein kinase (PRKAR1A): phenotype analysis in 353 patients and 80 different genotypes. J Clin Endocrinol Metab..

[244] Carney JA, Lyssikatos C, Seethala RR, Lakatos P, Perez-Atayde A, Lahner H, Stratakis CA (2018). The Spectrum of Thyroid Gland Pathology in Carney Complex: The Importance of Follicular Carcinoma. Am J Surg Pathol..

[245] Stratakis CA, Raygada M. Carney Complex. 2003 Feb 5 [updated 2018 Aug 16]. In: Adam MP, Ardinger HH, Pagon RA, Wallace SE, Bean LJH, Stephens K, Amemiya A, editors. GeneReviews® [Internet]. Seattle (WA): University of Washington, Seattle; 1993–2020. 20301463

[246] Koopman RJ, Happle R (1991). Autosomal dominant transmission of the NAME syndrome (nevi, atrial myxoma, mucinosis of the skin and endocrine overactivity). Hum Genet..

[247] Rhodes AR, Silverman RA, Harrist TJ, Perez-Atayde AR (1984). Mucocutaneous lentigines, cardiomucocutaneous myxomas, and multiple blue nevi: the "LAMB" syndrome. J Am Acad Dermatol..

[248] Carney JA, Sheps SG, Go VL, Gordon H (1977). The triad of gastric leiomyosarcoma, functioning extra-adrenal paraganglioma and pulmonary chondroma. N Engl J Med..

[249] Settas N, Faucz FR, Stratakis CA (2018). Succinate dehydrogenase (SDH) deficiency, Carney triad and the epigenome. Mol Cell Endocrinol..

[250] Correa R, Salpea P, Stratakis CA (2015). Carney complex: an update. Eur J Endocrinol..

[251] Hattori S, Yamane Y, Shimomura R, Uchida Y, Toyota N, Miura Y, Shiota S, Tajima Y (2018). Carney complex: a case with thyroid follicular adenoma without a PRKAR1A mutation. Surg Case Rep..

[252] Young WF Jr, Carney JA, Musa BU, Wulffraat NM, Lens JW, Drexhage HA. Familial Cushing's syndrome due to primary pigmented nodular adrenocortical disease. Reinvestigation 50 years later. N Engl J Med. 1989;321(24):1659–64. 10.1056/NEJM198912143212407 2586567

[253] Mete O, Asa SL (2012). Pitfalls in the diagnosis of follicular epithelial proliferations of the thyroid. Adv Anat Pathol..

[254] Asa SL (2020). Survival Guide to Endocrine Pathology.

[255] Mete O, Boyce AM, Stratakis CA, Weinstein LS, Lloyd RV, Osamura RY, Klöppel G, Rosai J (2017). McCune-Albright syndome. WHO Classification of Tumours of Endocrine Organs.

[256] Nwokoro NA, Korytkowski MT, Rose S, Gorin MB, Penles Stadler M, Witchel SF, Mulvihill JJ (1997). Spectrum of malignancy and premalignancy in Carney syndrome. Am J Med Genet..

[257] Halászlaki C, Takács I, Butz H, Patócs A, Lakatos P (2012). Novel genetic mutation in the background of Carney complex. Pathol Oncol Res..

[258] Courcoutsakis N, Patronas N, Filie AC, Carney JA, Moraitis A, Stratakis CA (2009). Ectopic thymus presenting as a thyroid nodule in a patient with the Carney complex. Thyroid..

[259] Stratakis CA, Carney JA, Lin JP, Papanicolaou DA, Karl M, Kastner DL, Pras E, Chrousos GP. Carney complex, a familial multiple neoplasia and lentiginosis syndrome. Analysis of 11 kindreds and linkage to the short arm of chromosome 2. J Clin Invest. 1996;97(3):699–705. 10.1172/JCI118467 PMC507106 8609225

[260] Oshima J, Martin GM, Hisama FM. Werner Syndrome. 2002 Dec 2 [updated 2016 Sep 29]. In: Adam MP, Ardinger HH, Pagon RA, Wallace SE, Bean LJH, Stephens K, Amemiya A, editors. GeneReviews® [Internet]. Seattle (WA): University of Washington, Seattle; 1993–2020. PMID: 20301687. 20301687

[261] Yokote K, Chanprasert S, Lee L, Eirich K, Takemoto M, Watanabe A, Koizumi N, Lessel D, Mori T, Hisama FM, Ladd PD, Angle B, Baris H, Cefle K, Palanduz S, Ozturk S, Chateau A, Deguchi K, Easwar TK, Federico A, Fox A, Grebe TA, Hay B, Nampoothiri S, Seiter K, Streeten E, Piña-Aguilar RE, Poke G, Poot M, Posmyk R, Martin GM, Kubisch C, Schindler D, Oshima J (2017). WRN Mutation Update: Mutation Spectrum, Patient Registries, and Translational Prospects. Hum Mutat..

[262] Goto M, Ishikawa Y, Sugimoto M, Furuichi Y (2013). Werner syndrome: a changing pattern of clinical manifestations in Japan (1917–2008). Biosci Trends..

[263] Takemoto M, Mori S, Kuzuya M, Yoshimoto S, Shimamoto A, Igarashi M, Tanaka Y, Miki T, Yokote K (2013). Diagnostic criteria for Werner syndrome based on Japanese nationwide epidemiological survey. Geriatr Gerontol Int..

[264] Lauper JM, Krause A, Vaughan TL, Monnat RJ (2013). Spectrum and risk of neoplasia in Werner syndrome: a systematic review. PLoS One..

[265] Goto M, Miller RW, Ishikawa Y, Sugano H (1996). Excess of rare cancers in Werner syndrome (adult progeria). Cancer Epidemiol Biomarkers Prev..

[266] Ishikawa Y, Sugano H, Matsumoto T, Furuichi Y, Miller RW, Goto M (1999). Unusual features of thyroid carcinomas in Japanese patients with Werner syndrome and possible genotype-phenotype relations to cell type and race. Cancer..

[267] Yu CE, Oshima J, Fu YH, Wijsman EM, Hisama F, Alisch R, Matthews S, Nakura J, Miki T, Ouais S, Martin GM, Mulligan J, Schellenberg GD (1996). Positional cloning of the Werner's syndrome gene. Science..

[268] Croteau DL, Popuri V, Opresko PL, Bohr VA (2014). Human RecQ helicases in DNA repair, recombination, and replication. Annu Rev Biochem..

